# BELMM: Bayesian model selection and random walk smoothing in time-series clustering

**DOI:** 10.1093/bioinformatics/btad686

**Published:** 2023-11-14

**Authors:** Olli Sarala, Tanja Pyhäjärvi, Mikko J Sillanpää

**Affiliations:** Research Unit of Mathematical Sciences, University of Oulu, FI-90014 Oulu, Finland; Department of Forest Sciences, University of Helsinki, FI-00014 Helsinki, Finland; Research Unit of Mathematical Sciences, University of Oulu, FI-90014 Oulu, Finland

## Abstract

**Motivation:**

Due to advances in measuring technology, many new phenotype, gene expression, and other omics time-course datasets are now commonly available. Cluster analysis may provide useful information about the structure of such data.

**Results:**

In this work, we propose BELMM (Bayesian Estimation of Latent Mixture Models): a flexible framework for analysing, clustering, and modelling time-series data in a Bayesian setting. The framework is built on mixture modelling: first, the mean curves of the mixture components are assumed to follow random walk smoothing priors. Second, we choose the most plausible model and the number of mixture components using the Reversible-jump Markov chain Monte Carlo. Last, we assign the individual time series into clusters based on the similarity to the cluster-specific trend curves determined by the latent random walk processes. We demonstrate the use of fast and slow implementations of our approach on both simulated and real time-series data using widely available software R, Stan, and CU-MSDSp.

**Availability and implementation:**

The French mortality dataset is available at http://www.mortality.org, the *Drosophila melanogaster* embryogenesis gene expression data at https://www.ncbi.nlm.nih.gov/geo/query/acc.cgi?acc=GSE121160. Details on our simulated datasets are available in the Supplementary Material, and R scripts and a detailed tutorial on GitHub at https://github.com/ollisa/BELMM. The software CU-MSDSp is available on GitHub at https://github.com/jtchavisIII/CU-MSDSp.

## 1 Introduction

The new and cheaper RNA sequencing methods have made gene expression time-course data more feasible. With RNA-seq, gene expression data can be obtained from thousands or tens of thousands of genes simultaneously. Gene expression data collected across time points can be used for studying, e.g. developmental processes and organisms’ molecular level responses to environmental changes. The diversity of biological questions, time scales and the number of measurements set challenges for creating generally applicable methods for analysing the time-series data. For now, the typical experimental setup is to collect 5–20 consecutive measurements from a small number of replicates while the number of genes is vast. This makes clustering-type methods highly desirable for dividing the data into smaller, homogeneous, and more manageable partitions, e.g. because the genes clustered together based on their expression time series patterns are likely co-regulated by the same transcription factors or external cues (Ramoni *et al.* 2002, Pyatnitskiy *et al.* 2014, Wollman *et al.* 2017).

While there are many algorithms and techniques for clustering data, the methods for gene expression time-course data should be flexible given the many experimental settings. Hence, it is worth considering mixture modelling as a starting point (McLachlan *et al.* 2019). One of the most well-known examples of a mixture model is the Gaussian mixture model. Assuming $X\in R^{n\times d}$ is a matrix representation of the time series dataset without missing values and without taking difference operation to make it stationary, its density can be modelled with a Gaussian mixture model (Frühwirth-Schnatter 2006, Eirola and Lendasse 2013) with probability density


(1) $$p(X|\theta )=\sum_{k=1}^{K}\pi_{k}N(X|\mu_{k},\Sigma_{k}).$$

Above, $N(X|\mu_{k},\Sigma_{k})$ is the probability density function of the multivariate normal distribution and *π*_1_,…,*π_K_* are the weights of the *K* components, also interchangeably called mixing coefficients, with a property


$$\sum_{k=1}^{K}\pi_{k}=1.$$

In the context of genes, these components refer to the underlying structure of the data, with observations (gene expression profiles) from the same component (cluster of genes) being similar. The weights give the probability that an observation is seen from one such structure, thus explaining homogeneity in the data.

The parameters in Equation (1) are traditionally estimated by maximizing the log-likelihood function by expectation–maximization (EM) algorithm or by Markov chain Monte Carlo (MCMC) due to complexity or lack of closed-form solutions (see, e.g. Albert 2009, Michael and Melnykov 2016). In terms of the Gaussian mixture model, the connection between mixture modelling and clustering can be considered as follows:

1. Mixing coefficients *π_k_* describe how much weight is given to the *k*:th mixture component in the mixture distribution (1). With a finite number of observations to assign, it also represents cluster size—the share of observations to be assigned to cluster *k*.
2. Mean vectors $\mu_{k}$ capture the overall trend curves of the corresponding mixture components. In the clustering context, they can be considered the centroids of the clusters.
3. Covariance matrices $\Sigma_{k}$ describe the dependencies within the observations in *k*:th mixture component. Depending on the application, the elements of the covariance matrices may include temporal and spatial variance components and measurement error. It is also quite common to assume the variance is constant for each mixture component through time. In the clustering context, the covariance matrices contain information on the variance within the clusters and explain the variance between them.

Time dependency in the observed data and other restrictions may be taken into account by specifying each of the mean vectors $\mu_{k}$ its own prior distribution, allowing rich model structures. Typically, these specifications include a smoothing effect, e.g. borrowing strength from both the neighbouring observations and time points (Xu *et al.* 2020), over the fitted model, which is particularly useful with noisy data. Smoothing can also reduce the number of parameters required to fit the model. For example, Golumbeanu *et al.* (2019) uses cubic smoothing splines in their implementation of (1) to cluster host genes by their temporal expression patterns as part of HIV-1 replication research. Alternatively, a widespread assumption for smoothing the data over *τ* adjacent time-points (t,…,t−τ) is the autoregressive AR(*τ*) process. Notably, the AR(1) process


(2) $$\mu_{k,t}=\rho_{1}\mu_{k,t-1}+\rho_{2},$$

where *ρ*_1_ and *ρ*_2_ are parameters controlling the shape of the process. The benefit of this choice is the greatly reduced complexity and the overall reduction in computational cost. Further simplification can be obtained by assuming stationarity, or zero-mean AR(1) process, $\mu_{k}=0$. With these assumptions, the covariance structure could fully describe the observations associated with each mixture component (see, e.g. Pourahmadi 2013). However, in the context of the model (1), the zero-mean assumption might only be appropriate for some applications. This comes with a more computationally costly estimation of the covariance.

Another interesting approach to clustering time-series data is to use the principal curve clustering method (Wu *et al.* 2021). Here, the data are first approximated by functional principal component analysis methods, and the time series are grouped by clustering the principal scores. The reduction in dimension gained through this approach allows Wu *et al.* (2021) to use a computationally heavier algorithm to not only cluster the scores but to choose the optimal number of clusters at the same time using Bayesian information criterion (BIC) (Schwarz 1978). In our opinion, while their method is fundamentally different, the clustering results of this approach are close to the cubic spline version of the Gaussian mixture model of Golumbeanu (2020) for the French mortality data.

Choosing the correct number of mixture components *K* is a fundamental problem in mixture modelling and has been studied extensively (see, e.g. Milligan and Cooper 1985, Tibshirani *et al.* 2001, van der Nest *et al.* 2020). Having too few or many components can hinder the model’s performance by leading to under and overfitted models, respectively. In addition, having excess components is wasteful since estimating additional parameters increases the computation time and could lead to some empty components. The most common approach is to use information criteria such as Akaike information criterion or BIC (van der Nest *et al.* 2020). Repeated runs of the EM algorithm for different values of *K* are required since it is susceptible to finding the local optima, which may not be the best fit for the whole data. In these cases, the speed of the convergence of the EM algorithm may become slow. Calculating some of the criteria for many different models can be challenging. Also, the inability to compare the values between some of the criteria for unnested models makes the model selection more difficult.

Another alternative to the information criteria is the Reversible Jump-MCMC (RJMCMC) proposed by Green (1995), which is a trans-dimensional sampling method that can be used for model selection and to find the plausible number of mixture components (Richardson and Green 1997). Many studies were conducted on this use-case, but being hard to implement and suffering from many problems such as bad mixing properties, high computational cost, and nonexploitation of parallel computing, make it impractical to use (see, e.g. Cappé *et al.* 2003, Frühwirth-Schnatter 2006). Some of these problems have recently been overcome in the highly parallelisable RJMCMC software implementation CU-MSDSp (Chavis *et al.* 2021). This software fully utilizes the new theoretical and technical developments around the RJMCMC (Chavis 2020, Chavis *et al.* 2021). Where the mixing and convergence properties of the original RJMCMC implementations are dependent on good proposal distributions between the models, the CU-MSDSp utilizes Bayes’ Theorem to calculate the model posterior distribution straight from the precalculated models, called ‘Gold-standard chains’. The models can be calculated in parallel, and the number of burn-in and sampling iterations can be set so that all the individual models are known to converge, saving computational resources. Similarly to the traditional RJMCMC, the best-fitting model can be chosen from the model posterior distribution. Compared to the EM-based approaches, RJMCMC enables easy evaluation of the fit of wildly differing model structures. For example, it is possible to choose between AR processes and different smoothing distributions (Cauchy versus Normal versus Student’s *t*). For an in-depth discussion on the model selection with RJMCMC, see Hastie and Green (2012).

This article is organized as follows: first, we present our proposed latent mixture model for estimating cluster centres from biological time-series data. Secondly, we consider model selection to decide the number of groups *K*. Third, we consider assignment and distance functions to cluster individual time series. Finally, we evaluate our method with two simulated and two real datasets and compare it with other methods. A discussion follows this.

## 2 Materials and methods

In this section, we go through the steps of the BELMM (Bayesian Estimation of Latent Mixture Models) approach; First, the mixture component centres are estimated using latent random walk processes. Then, the most plausible model is selected using RJMCMC. Last, the assignment of observations to the centres is done after the model selection.

Like before, we assume $X\in R^{n\times d}$ is a data matrix without missing values. Instead of the model (1), we can consider a more general mixture distribution given by


(3) $$X|\theta ∼\sum_{k=1}^{K}\pi_{k}f_{k}(X|\theta_{k})=\sum_{k=1}^{K}\pi_{k}\phi_{k},$$

where $f_{k}(.)$ is some probability density corresponding to the *k*:th mixture component conditioned on a finite set of parameters θ, *K* being the number of mixture components. Here we have written all the parameters $\theta =(K,\pi_{1},\ldots ,\pi_{K},\theta_{1},\ldots ,\theta_{K})$ under one symbol, but it is not required the functions *f_k_* depend on all of them.

### 2.1 Bayesian latent process approach

We detailed the traditional method of estimating mixture components in the introduction. Our approach treats the time-series data as an outcome of independent draws from some latent random walk processes. If we assume one such process corresponds to each mixture component $\phi_{k}$, the original task of fitting model (3) is transformed into the estimation of *K* random walk processes, ${\left\{z_{k}\right\}}_{k=1}^{K}$. On the other hand, we can treat the individual observed time series from each mixture component as i.i.d., the benefits of which we discuss later. For clarity, let us assume the random walk process is Gaussian. We note that it could be based on other probability density functions, say Cauchy or Student’s *t* distributions. For further details on using random walk prior for smoothing and their connection to penalized likelihood estimation, we refer the reader to Lang and Brezger (2004).

Now, the goal with the estimation is to find parameter values for $z_{k}$ such that the sample draws $z_{k}=(z_{k,1},\ldots ,z_{k,d})\in R^{d}$, where for time points t∈(1,…,d)


(4) $$z_{k,t}∼N(z_{k,t-1},\varepsilon_{k}^{2}),$$

the individual observations $x_{i}$ follow the law of, now doubly stochastic process


(5) $$x_{i}∼\sum_{k=1}^{K}\pi_{k}N(z_{k},\sigma_{k}^{2}).$$

In the case of the Gaussian random walk, the set of hyper-parameters is $\theta_{k}=(z_{k,0},\varepsilon_{k}^{2},\sigma_{k}^{2})$, for which we can set suitable prior distributions.

From the formula for the random walk (4), we see that the smoothness of the latent processes depends on the parameter $\varepsilon_{k}^{2}$. We can use this by setting scale-dependent empirical priors on $\varepsilon_{k}^{2}$ or fixing them beforehand accordingly. The formulation enables us to estimate mixture components with varying levels of smoothness. In this work, we used prespecified, data scale-dependent values of $\varepsilon_{k}^{2}$ to speed up convergence. The details can be found in the Supplementary Material.

Our choices lead to a posterior distribution of the latent variable model


(6) $$\begin{array}{c} p(\theta |X)=\\ \frac{1}{p(X)}\sum_{k=1}^{K}\left[\prod_{i=1}^{n}p(x_{i}|\pi_{k},z_{k},\sigma_{k}^{2})\right]\prod_{k=1}^{K}\left[p(z_{k}|z_{k,0},\varepsilon_{k}^{2})p(\sigma_{k}^{2})\right], \end{array}$$

where


$$p(z_{k}|z_{k,0},\varepsilon_{k}^{2})=p(z_{k,0})\prod_{t=1}^{d}p(z_{k,t}|z_{k,t-1},\varepsilon_{k}^{2})$$

is the prior of the random walk process (4). The log posterior of the model is


(7) $$\begin{array}{c} \;\text{log}(p(\theta |X))\propto {\text{LSE}}_{k=1}^{K}(\sum_{i=1}^{n}\;\text{log}\;\left(p(x_{i}|\pi_{k},z_{k},\sigma_{k}^{2})\right))\\ +\sum_{k=1}^{K}\left[\text{log}\;\left(p(z_{k}|z_{k,0},\varepsilon_{k}^{2}))+\text{log}(p(\sigma_{k}^{2})\right)\right], \end{array}$$

where ${\text{LSE}}_{k=1}^{K}(y_{k})=\text{log}\;\left(\sum_{k=1}^{K}\;\text{exp}\;(y_{k})\right)$ is the LogSumExp function. Last, it is worth noting that in terms of covariance, for each mixture component, we effectively have $\Sigma_{k}=I\cdot \sigma_{k}^{2}$.

### 2.2 Model selection and the number of components *K*

Specifying the correct number of mixture components is important for the computation and interpretability of a model. This subsection gives an overview of how we approached this challenge. A detailed description of the process is given in Supplementary Section S3.

We begin by making an educated guess of the number of components based on prior information, such as the number of treatments. Without prior knowledge, the alternative would be to consider the number of mixture components *K* as a random variable for which a prior range can be obtained with a tool such as the R package NbClust (Charrad *et al.* 2014). Next, we write the corresponding models $M_{1},M_{2},\ldots ,M_{J}$, where each model $M_{j}$ is structured like in Section 2.1. Then, we can determine the most plausible model using the RJMCMC implemented in the software CU-MSDSp. The software does MCMC estimation of the Bayesian models using Stan and calculates a model posterior distribution from which the most probable model can be identified. If the models $M_{1},M_{2},\ldots ,M_{J}$ are nested, and empty components are observed after the assignment step, it is possible to marginalize the model posterior distribution on the number of nonempty components (cf. van Havre *et al.* 2015). We call this rectified model posterior distribution. Again, it can be used to select the most plausible model. For more details, see Supplementary Section S2.

### 2.3 Assignment

Until now, we have only considered the modelling of cluster centres. In this subsection, we go through the supervised task of assignment operation: how the observations (e.g. individual time series per gene) can be assigned to predefined centres and known clusters, e.g. classifying genes into clusters showing specific expression patterns over time.

After estimating the posteriors of the latent-variable models, we can use the found mixture components to form the clusters. Distances between individual observations and all cluster centres are determined. We can assign each observation to the cluster with the shortest distance. The distance can also be converted to a probability scale [see Equation (8)]. This approach comes with three benefits. First, the assignment can be done independently of the MCMC simulations, and there is no need to repeat all calculations if one wants to change the distance function. Also, this reduces computational burden (MCMC updating) and avoids label switching since there are no cluster membership variables to be updated (for label switching, see references in van Havre *et al.* 2015). Secondly, this makes it possible to easily adjust the threshold probabilities for assigning the individual observation to some clusters and finding the strong memberships (cf. Hautamäki *et al.* 2005). Hence, it is possible to exclude certain kinds of observations (e.g. noisy observations) from clusters, which can benefit some applications, see Discussion. Conversely, any additional or new data can also be assigned to pre-existing clusters (cf. predict. Mclust, Scrucca *et al.* 2016). Using any distance function for this step is the third and possibly greatest benefit. Thus, it is possible to create custom distance functions specifically for one’s application or pick up one of the 46 measures available in R package philentropy (Drost 2018). For the assignment step in the examples of this article, we have used


(8) $$p(x_{i}\in k|\theta_{k})\propto \frac{\pi_{k}}{\sigma_{k}}\text{exp}\;\left(-\frac{||z_{k}-x_{i}|{|}^{2}}{2\sigma_{k}^{2}}\right).$$

After the labels are given, it is possible to calculate realized values as secondary estimates for the mixture weights. These values can be used to further validate the method to see if the estimated mixture components are consistent with the assignments.

## 3 Validation analyses on simulated datasets and an empirical analysis

We demonstrate the use of the BELMM on two simulated and two real datasets so that a meaningful comparison can be made with analogous methods.

### 3.1 Simulated datasets

First, we have the toy-example data from the R package TMixClust. It consists of a 91 time series belonging to three clusters that were simulated according to a mixed-effects model


$$x_{i,j}=\xi_{k}(t_{j})+\beta_{i}+\varepsilon_{i,j},$$

where $\xi_{k}(t_{j})$ is a cluster-specific fixed effect following some cubic polynomial, *β_i_* a random effect corresponding to the gene effect, and $\varepsilon_{i,j}$ a random measurement error (Golumbeanu 2020). For this dataset, our primary focus is on the fit of the model (6) compared to the cubic splines approach. With this dataset, we studied the effect of the smoothing prior (4) under four different assumptions about the mixture distribution, *f_k_*. These were the Gaussian, Cauchy, Laplace, and Student’s-*t* distributions. We compared the fit of these models to the data using RJMCMC. For additional comparison, random walks with AR(1) model mean (2) were also considered, again under the different mixture distributions.

The second simulated dataset is a collection of five different time-series datasets with K=1,…,5. Each dataset consists of a 100 series drawn from a set of linear and nonlinear functions, forming clusters of different sizes. With these datasets, we aimed to see how our proposed random walk method could identify the different mixture components as the amount of data from each component got proportionally smaller as *K* increased. The results were then compared to the ones obtained by TMixClust. We also wanted to see how well the RJMCMC distinguishes the correct number of components. For each of the five datasets, we estimated eight models following the details in Section 2.1. The only differentiating factor between the models is the number of mixture components; the model $M_{1}$ with one component, model $M_{2}$ with two components, and so on. We compared the resulting model posterior distribution to the number of clusters found using the mixture components as outlined in Section 2.3.

### 3.2 French mortality

The third time-series dataset is a yearly French mortality data for 1816–2018 (Human Mortality Database 2020). The data consists of mortality rates of age groups 0–109+. For this empirical study, we used log-transformed mortality rate data for age groups 0–101 from all the available years. We excluded the data for the oldest age groups because it was unavailable for all the years. We chose this dataset to compare the results with those from functional data clustering (Wu *et al.* 2021). Also, this data allows us to study the scalability of the random walk process as the data can be used in two ways: either as 203 profiles of length 101 (the one used by Wu *et al.* 2021) or 101 time series of length 203. Regardless of which of these we consider, the number of parameters in the model increases rapidly together with the number of mixture components. For this data, we estimated five and eight models formulated according to Section 2.1 with a different number of mixture components in each.

### 3.3 *Drosophila melanogaster* embryogenesis gene expression

To test the methodology with actual empirical gene expression data, we use the fourth dataset, the *Drosophila melanogaster* embryogenesis gene expression data (Becker *et al.* 2018). The data consists of 14 consequent measurements of 17 558 genes from four biological replicates. As the level of expression for the genes, for each time point, we calculated the average of the log2-transformed counts of the replicates. These were weighted such that the relative number of ambiguous or failed reads was the same between all the individual measurements.

The raw gene expression data may contain, e.g. measurement artefacts or other systematic anomalies that do not correspond to any biological process (Ma and Kingsford 2019). On the other hand, the data are actual measurement data, and it is only sometimes possible to analyse already processed data. With that in mind, we first approach the data blindly and use all of it. Then, we apply thresholding to limit the effect of noise in the data and to obtain smaller clusters.

Since we did not have an actual prior range for the number of components, we used the silhouette index with Ward’s method implemented in NbClust (Charrad *et al.* 2014) to the full data, which suggested the number is low (Fig. 3). Hence, we focused on models with one to eight mixture components.

## 4 Results

In this section, we give short summaries of the results of the four analyses. When referring to the estimated value, we mean the posterior median values. For the simulated datasets, we evaluated the fit of the parameter estimates with mean squared error (MSE). As the mixture model often suffers from between-chains label switching, the expression ‘best fitting model’ refers to the model which provides the smallest MSE to the simulation parameters over permutations. The R scripts for the data simulation and processing are on the GitHub page. Information on the fast and slow implementations of the model is provided in Supplementary Section S6.

### 4.1 Simulated datasets

The detailed results discussed from the BELMM approach and TMixClust can be found in Supplementary Section S3. The MSE values are given for the model with the number of components that were used in the simulation of the data.

RJMCMC provides posterior probabilities for different smoothing models conditionally on the data, which allows comparisons among nonnested models. Based on these probabilities, we can make the following conclusions: Other than runtime, we did not observe the assumption about the mixture distribution to play a considerable role in the toydata (Supplementary Fig. S5a). The matched cluster centres were approximately the same shape (MSE = 0.074, within the estimated values), with the mixture components differing slightly in their variance parameters. This was also suggested by the RJMCMC, which resulted in an almost even distribution between the models (Supplementary Fig. S5b). With the AR processes as the mean, we did observe a difference between the models (Supplementary Fig. S6a). While the estimated centres were mostly equal (MSE = 13.97, within the estimated values), the longer-tailed distributions were favoured over the normal distribution by the RJMCMC due to AR(1)’s looser fit to the data (Supplementary Fig. S6b).

For inferring the number of components in the simulated datasets, the RJMCMC favoured models with more components for all the simulated datasets (see e.g. Supplementary Figs S8, S11, and S17). Correcting the model posterior distributions from the empty components, the rectified model posterior distribution always supported the number of components we used in the data simulation. Hence, one can say that the BELMM approach correctly identifies the structure of these datasets, but it cannot be trusted blindly.

Comparing the clustering results from the BELMM approach with TMixClust, both methods perform equally well when the number of clusters in the analysis stage is set smaller or equal to the simulated number (Supplementary Tables S1 and S2). Otherwise, the spline-based algorithm splits the components into smaller ones, while our approach leaves some clusters empty. The tendency of not forcing observations into additional components seems beneficial, but we cannot guarantee it happens for all the datasets.

### 4.2 French mortality

With the French mortality data, we found that the analysis worked well in both interpretations of the data, but there was a significant difference in runtime between the 101 and 203 time points (Supplementary Section S4). Comparing the estimated and realized mixture weights shows that the assignment worked consistently through the different analyses (Supplementary Tables S8 and S11).

**Figure 1.**
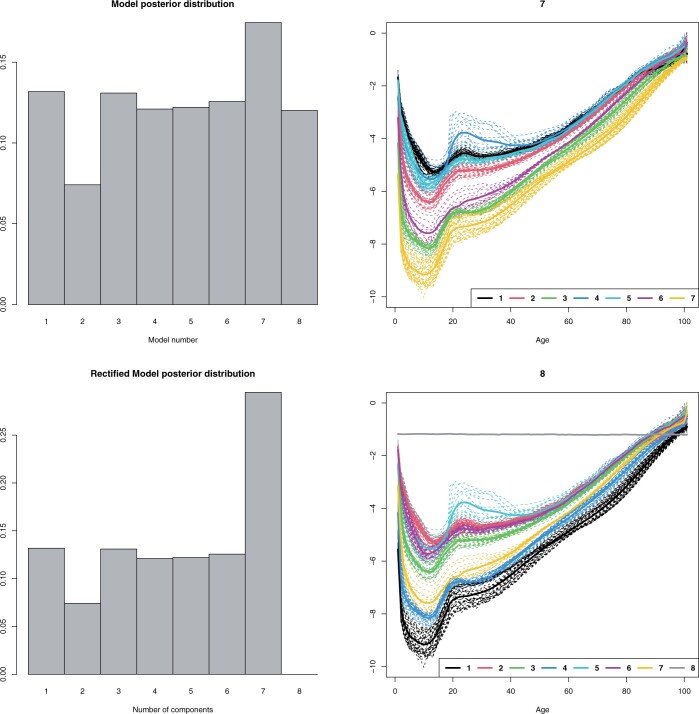
The French mortality, *d *=* *101. On the left column are the model posterior distribution and the rectified model posterior distribution. On the right column are the estimated mixture component centres and realized clusters given by the slow implementation of the BELMM approach with fixed smoothing priors $\varepsilon_{k}^{2}=0.15,k=1,\ldots ,8$.

When considered as curves, *d *=* *101 and with two to five models, the resulting clusters were comparable to those found by the PCA-based functional curve clustering method (cf. Wu *et al.* 2021, Fig. 4 and Supplementary Fig. S26). Depending on the number of models and the type of implementation, we saw elevated posterior probability for either four or one and four components (Supplementary Fig. S23). With eight models at a time, we saw elevated probability for seven components (Fig. 3). The model with eight components resulted in an empty component, indicating the data could be fully described with seven components. This gave us the rectified model posterior distribution supporting the seven-cluster solution (Fig. 1). Applying TMixClust to the French mortality data resulted in different clusters than the BELMM approach. The main difference was the occurrence of empty components, which were not observed with TMixClust.

**Figure 2.**
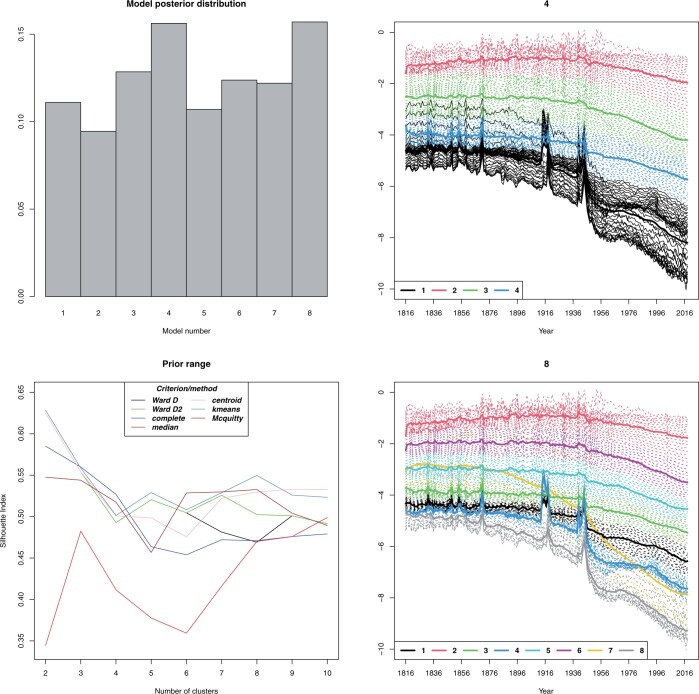
French mortality, *d *=* *203. On the left column are the model posterior distribution and the silhouette index given by the package NbClust using the noted methods guiding the decision of the prior range. On the right column are the estimated mixture component centres and realized clusters given by the slow implementation of the BELMM approach with fixed smoothing priors $\varepsilon_{k}^{2}=0.15,k=1,\ldots ,8$.

When we change to the time-series interpretation, *d *=* *203, there are no results to compare ours with. If we keep increasing the number of components, the data splits into smaller clusters. This indicates there is no clear cluster structure in the data. Having five models under consideration simultaneously, the most supported model had four mixture components, while with eight, the model posterior distribution shows signs of multimodality, suggesting that the data could be explained equally well with four or eight components (Fig. 2). A more in-depth analysis of the results is found in Supplementary Section S4.

### 4.3 *Drosophila melanogaster*

With the whole dataset, the estimation of the cluster centres was hindered by the least expressed genes. These observations attracted the random walk processes, leaving too few free mixture components for the rest of the data, slowing down the MCMC sampling (data not shown). After completing the first model estimation, we removed the genes not counted once in any replicates, allowing further analysis without the abovementioned issues. Those formed the first cluster, leaving us with 15 927 of the 17 558 genes (Supplementary Fig. S31).

**Figure 3.**
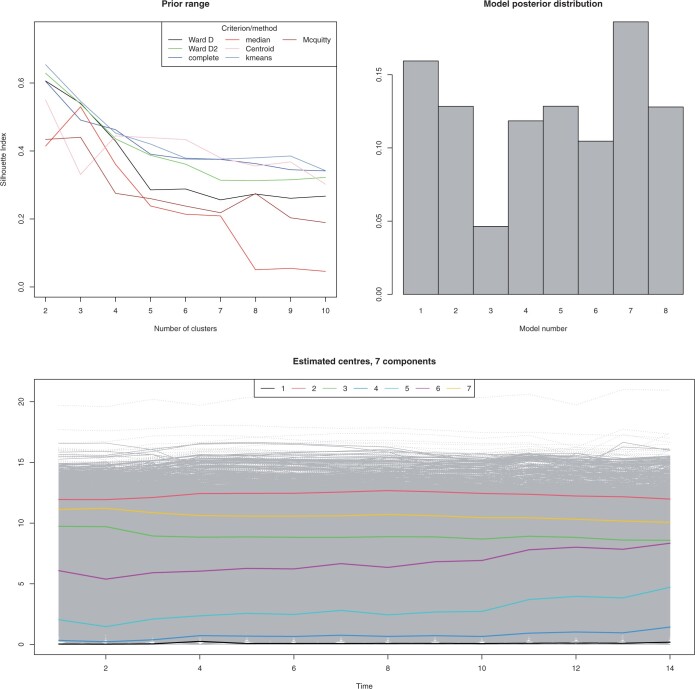
*Drosophila melanogaster* embryogenesis. On the top row: The silhouette index given by the package NbClust using the noted methods guiding the decision of the prior range and the model posterior distribution for the slow implementation with varying values for $\varepsilon_{k}^{2}$, respectively. On the bottom: Estimated mixture component centres given by the slow implementation of the BELMM approach with component-specific fixed values (0.1,0.47,0.75,1,1.3,1.7,2,2.5) for $\varepsilon_{k}^{2},k=1,\ldots ,8$.

Repeating the process on the zero-free data, RJMCMC estimation showed elevated posterior probabilities to the model with seven components (Fig. 3). However, the model posterior distribution shows signs of multimodality with additional peaks, indicating the data supports the models with fewer components. The model posterior distribution agrees with some of the methods used to calculate the silhouette index but is distinctly different from some of them (Fig. 3). Following the RJMCMC, we assigned the individual genes to seven clusters based on the parameter estimates within the most favoured model. Given the number of genes, the clusters were still quite large, so we applied thresholding to limit their size and make the analysis of the profiles more straightforward (results shown for the four-component model, Supplementary Fig. S35 and Supplementary Table S14).

The analysis of random subsets of genes, sized 1000 and 2000, showed the BELMM approach could estimate the mixture weights and centres comparably to the entire dataset (Supplementary Tables S14, S17, and S18). The model posterior distribution favoured the models with four and six components consistently with both fast and slow implementations of the model (Supplementary Figs S27, S37, and S40).

**Figure 4.**
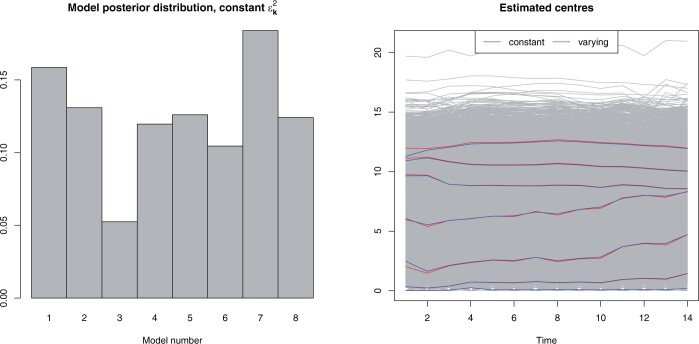
*Drosophila melanogaster* embryogenesis. On the left: Model posterior distribution for the fast implementation of the BELMM approach with constant values for $\varepsilon_{k}^{2}=0.1,k=1,\ldots ,8$. On the right: Estimated mixture component centres for the fast implementation with constant and the slow implementation with component-specific fixed values (0.1,0.47,0.75,1,1.3,1.7,2,2.5) for $\varepsilon_{k}^{2},k=1,\ldots ,8$.

We saw that the model posterior distribution depended on the choice of smoothing priors $\varepsilon_{k}^{2}$. Choosing values that were close to the estimated standard deviation resulted in the model gaining additional support if the order of the priors was changed. However, their effect was negligible if the values were chosen small enough. Comparing the model posterior distributions shown in (Figs 3 and 4), the differences are minimal (at the third decimal place). Hence, we recommend using a constant value of $\varepsilon_{k}^{2}$ for all the components to make analysing the model posterior distribution easier and to have the models nested for the possibility of rectification.

## 5 Discussion and conclusions

This article shows how time series clustering can be approached through random walk smoothing in a Bayesian setting. In addition, we have demonstrated how posterior distributions for parameters of these processes can be estimated through MCMC methods with simultaneous model selection based on the RJMCMC and how they can ultimately be used in supervised cluster assignment. The functionality of the proposed Bayesian method has been illustrated with simulated and actual empirical data.

### 5.1 Philosophy of model design: mean or covariance?

When designing a hierarchical structure for Bayesian mixture models, the statisticians have practically two fundamental ways to approach the problem with different assumptions; building a model either on a mean or on a covariance. When modelling through the mean, it is common to assume that each component’s covariance (variance) matrix is constant. With the covariance, the mean (expected value) is assumed to be constant, usually zero. In the mean-based methods, the estimation focuses on the shape of the data, issuing the deviation from it to a random chance according to the constant variance. This leads to a mixture of components with well-defined locations where the nearby observations share a high probability with that component. When modelling based on covariance, the estimation is usually through one or the product of kernel functions. The deviation from the mean is based solely on the covariance structure, which contains the random variation. As a result, the strong members of a mixture component share a common profile but not necessarily the same mean value. Both approaches have their benefits, but a compromise is made in the generality regarding the method’s applicability. We have observed that the mean-based approaches are more common in literature, and many examples were provided in the introduction. For example, methods based on covariance estimation are considered by Pigoli *et al.* (2014), Lee *et al.* (2015), Peng *et al.* (2019), Tavakoli and Panaretos (2016), and Masarotto and Masarotto (2023). We feel there is room for a rigorous discussion about the dichotomy of model assumptions in the clustering and mixture modelling literature.

Our proposed method focuses heavily on modelling the mean instead of the covariance. We use the random walk approach to estimate the mean, which allows us to capture a wide range of profiles in the time series data. To simplify the model, we assume the observations are independent draws from independent random walking processes, i.e. the time series corresponding to a single process is i.i.d. This gives us a model with fewer parameters to estimate, leading to more efficient calculations. The implication of our choices is a model that does not use all available information within the data traditionally captured by the covariance matrix in estimating the mixture components. We note this could affect forecasting/trend prediction and, in part, variance estimation.

Now, how significant the impact of simplifying the covariance estimation is? First, the individual observations have a combined effect on the estimation of latent processes, so the group-level variability is indirectly considered. Secondly, there is the assignment step, where the distances of the observations to the medians of the processes, or the centroids of the mixture components, are measured. Hence, the observations have a combined effect on the final result. Together, the methodology has a notable resemblance to the role of covariance in mixture modelling. In this case, the estimation is done at a different location than usual.

### 5.2 Special considerations

The latent processes we are considering have a smoothing effect on the data. When estimating the process’s mean, i.e. the centre of the mixture component, we find a profile representing the observations associated with that component. Suppose these centroids are used for future analysis. In that case, we can also consider this step as data aggregation and could be used as a dimension reduction operation for some higher dimensional applications. For example, if the centroids were formed from random subsets of the data, we saw the assignment step could be used to cluster the whole data with good accuracy. This is somewhat close to the idea Darkins *et al.* (2013) use for accelerating Bayesian hierarchical clustering. As for forecasting, one would expect the prediction accuracy with random walk behaviour to be relatively poor. However, it is widely used for noisy or otherwise unpredictable data and performs well in short-term forecasting of, e.g. weather and stock value (Nakamura and Small 2007). For longer-term forecasting, one can apply sequential Bayesian filtering, or a Kalman filter, to each cluster as a post-processing step and obtain accurate and efficient predictions (see, e.g. Doucet *et al.* 2000, Särkkä 2013).

### 5.3 Indicator variables

We have formulated our model without group membership indicator variables (labels) and thus avoided their MCMC estimation. Historically, their use has been a typical choice for modelling and estimating the mixture weights, see e.g. software package STRUCTURE (Pritchard *et al.* 2000). However, the Bayesian framework enables direct estimation of the mixture weights. These alternative ‘leave-out’ models based on marginalization correspond to the posterior from which the group membership has been analytically integrated out (cf. Stan User’s Guide, Chapter 7, Stan Development Team 2020). This works since the unknown mixture weights can be considered as random variables, with the restriction they sum up to one, and they are estimated just like the other unknowns in the model. The benefit of the latter approach depends on how the mixture weights were estimated using the labels. For this, let us consider two options where the mixture weights are directly estimated from the labels.

1. The first approach is treating the group membership indicators as a random variable. Then, for each iteration of the MCMC, the parameters of the corresponding mixture distributions are estimated based on the labelling. For a moderate number of components, this is fast. However, this approach is prone to within-chain label switching and requires many MCMC iterations for the model to converge fully.
2. The second approach is determining the group membership indicators as a function of the mixture densities. That is, estimating the individual mixture components from the data first. Then, labels for the individual observations are assigned based on the components on every iteration of the MCMC. This method is more robust than the above but requires more computation for each iteration of MCMC.

In both cases, the estimated mixture weights directly depend on the observed data. However, we can see that compared to the leave-out model; the second approach leads to redundant calculations.

We can now see why the marginalized approach feels appealing, but there is a catch. The estimated weights are now fully probabilistic and connected to the individual observations only by their likelihood, given the estimated mixture components. This should be computationally more efficient than having the indicators. However, while the resulting models are different, neither of the approaches is inherently better or worse than the other from the theoretical point of view. In our case, including the indicators would not drastically affect the estimation of the centroids of the mixture components since the individual observations will have an effect in either case.

### 5.4 Thresholding

We considered thresholding an interesting way to strengthen or lower the requirement for cluster membership probabilities as a preprocessing step and a further data visualization method. We found thresholding beneficial when working with *D.melanogaster* measurement data. We could identify and remove many genes acting like artefacts or noise with the raw data. After the initial run, we could fine-tune the cluster size using thresholding, improving the analysing process. Overall, thresholding is turning out to be a highly flexible process. It can be used for the whole dataset or some individual clusters. Hence, it is possible to justify removing genes with biologically irrelevant or outlier-like behaviour based on the deviation from the probabilistic model. The current thresholding implementation requires manual labour, but the clusters are ready for further analysis. Our approach is more sensible than count-based thresholding, say, removing all genes with <20 counts on average. It could be there are some genes expressed at those low levels, but of course, they could only be noise compared to genes with expression at tens or hundreds of thousands of counts. Given how thresholding worked for us, we see it as a valuable addition to the assignment step.

### 5.5 Reversible-jump MCMC

Last, we discuss RJMCMC as part of the model selection. Our simulated datasets show that the current implementation of the RJMCMC can be used to find the most plausible number of mixture components to a given data. Leaving out the birth-and-death processes, the current implementation of the RJMCMC is easier to compute, and the individual models are guaranteed to converge with shorter Markov chains as before. The disadvantage is that the mixture components with none, or just a few observations, are left alive, affecting the posterior probabilities of models. This complicates interpreting the model posterior distribution, hindering its intended use in aiding the analysis. One potential fix to this issue is to set an informative prior distribution favouring simpler models, which gives a higher probability for a smaller number of components. This parsimonious prior is well defined, but then one has to argue why, a priori, the number of components should be small, which is a highly subjective choice.

By our simulated data, we propose a rectified model posterior distribution where the support of the models with the false components is transferred to the model with a matching number of nonempty components. This way, we can afterwards correct inaccuracies in the model posterior distribution. However, one obvious issue with the rectified model posterior distribution is that the models are not guaranteed to have any empty components. We think this is due to the smoothness of the observations. Without much overlap between the individual series, plenty of which is in the simulated datasets, the conditions are sufficient for the clusters to keep splitting into smaller ones. Given the uniform prior, if there is no evidence for a smaller number of components, the higher number will always fit better than the smaller one. For these reasons, we think the current implementation of the RJMCMC with uniform priors is more suitable for comparing different smoothing models than inferring the number of components. Otherwise, we would treat it as a measure of clusterability. Then again, the performance of the RJMCMC depends on the models in question. Our smoothing models may not best suit all these datasets.

## Supplementary Material

btad686_Supplementary_Data
