# Supplementary material for: BELMM: Bayesian model selection and random walk smoothing in time-series clustering
Source: Bioinformatics. 2023 Nov 14;39(11):btad686. doi: 10.1093/bioinformatics/btad686 (PMC10686958; doi:10.1093/bioinformatics/btad686)
Supplement: btad686_Supplementary_Data [file btad686_supplementary_data.pdf]

`BELMM`: Bayesian model selection and random walk smoothing in time-series clustering

Supplementary material

Olli Sarala, Tanja Pyhäjärvi and Mikko J. Sillanpää

April 2023

Contents

1 Foreword **2**

2 Reversible jump MCMC **2**

3 Simulation study **4**
- 3.1 TMixClust Toydata 4
  - 3.1.1 Comparison of smoothing distributions 8
- 3.2 Simulated data sets 10
  - 3.2.1 Single component 11
  - 3.2.2 Two components 13
  - 3.2.3 Three components 16
  - 3.2.4 Four components 18
  - 3.2.5 Five components 21

4 French mortality **23**
- 4.1 Interpretation 1: 203 profiles of length 101 23
- 4.2 Interpretation 2: 101 time series of length 203 28

5 *Drosophila melanogaster* embryogenesis time-series data set **32**
- 5.1 Analysis on full data 32
- 5.2 Analysis on random subsets 36
- 5.3 Influence of starting values on the posterior estimates 46

6 Appendix: Software details **46**
- 6.1 `BELMM` 46
- 6.2 `R` 47
- 6.3 Stan 47
- 6.4 CU-MSDSp 48
- 6.5 `TMixClust` 49
- 6.6 Software versions 49

1 Foreword

This supplementary is organised as follows: First, we complement the discussion regarding model selection with the Reversible jump MCMC, followed by explaining the rectified model posterior distribution. Then, we provide detailed results from the simulation studies and the two empirical analyses. All the `R` code for producing the figures and tables is available in an accompanied `R` file with code for data simulation and processing (on GitHub at `https://github.com/ollisa/BELMM`). The actual time series data sets are not included (French mortality data due to license agreement, *Drosophila melanogaster* due to copyright concerns). Still, they can be downloaded free from their original repositories, the French mortality data `http://www.mortality.org/` and *Drosophila melanogaster* `https://www.ncbi.nlm.nih.gov/geo/query/acc.cgi?acc=GSE121160`. The "results" files from `CU-MSDSp` are also not included since they are large and contain the aforementioned data sets.

Why is the method called "latent"?

This wording refers to a more general "latent variable models" concept. In short, "latent" comes from the model assumption that some unobserved parameters influence the observed data.

2 Reversible jump MCMC

Additional discussion points

The RJMCMC approach has some notable advantages over the information criterion-based counterparts. First, we have a high level of flexibility regarding model structures we can compare. The models need be nested, especially when we only want to determine the number K, but unnested models can also be compared. With RJMCMC, it is possible to compare models where each of the mixture components have their own model structures. Between the models, each of the mixture components can also have their own set of priors, say a mixture of Gaussian and Laplace distributed components or a mixture of components with different levels of smoothing in each, if we want some of the components to have more variation than others. Second, we obtain the model posterior distribution, not just a list of values for the different information criteria. This allows us to make sensible comparisons between apples and oranges -situations. It can also be beneficial when there is no clear-cut solution to the application at hand. However, as a drawback, there is a higher computational burden with the RJMCMC approach.

Last, we have left open the interpretation of the model posterior distribution. In the traditional RJMCMC, if the only difference between the models were the number of components K, the model posterior distribution would be the posterior distribution of the number of components, $P(K|\text{Data})$. However,

CU-MSDSp neither utilises the Birth-and-Death nor the Birth-Combine-Death process, on which the traditional methods are based (see, e.g. [Cappé et al., 2003]). This means that the implementation of RJMCMC does not penalise the models with false or loosely supported components, which can skew the model posterior distribution.

On the model level, the obvious first aid would be setting the prior range for the number of mixture components such that the weights of the false components are restricted, a priori, to zero; Any models with false components could then be left out of the RJMCMC altogether. This would require more work before the actual model selection, somewhat defeating the purpose of using RJMCMC. Also, another hit to the objectivity of the model selection arises from the 'curse of dimensionality'. Theoretically, it makes sense to avoid the false components. However, even with a restrictive prior structure, the estimated weights will be small but never truly zero. Hence, if the number of observations is sufficiently large, some will be 'erroneously' assigned to the loosely supported clusters. Then one would need to remove these models with seemingly possible mixture components from the model selection without observing their effect on the model posterior distribution.

On the RJMCMC level, a way to solve this problem would be to set a more informative (restrictive) model-prior distribution, favouring the solutions with fewer components. In the current implementation of the CU-MSDSp, the prior is set to flat [Chavis et al., 2021], a neutral/uninformative choice. We note that changing the prior could limit the applicability and generality of RJMCMC. It may even be a beneficial choice when many models are estimated simultaneously since the probability of each will likely be small from the start. Hence, the chance of the model with falsely supported components "dying" (the probability in the model posterior distribution approaching 0) should increase. Our examples did not show this behaviour, but it opens an interesting research topic for the future (cf. [van Havre et al., 2015]).

Rectified model posterior distribution

Considering the above, our approach to solving the occurrence of empty components is to form a rectified model posterior distribution whenever false components are present (with respect to the assignment rule). We do this as a post-processing step by marginalising the model posterior distribution on the number of non-empty components. Still, an equally good alternative would be considering all components with weights falling under the given threshold as 'false'. In practice, we do the marginalisation by transferring the amount of support the model with false components gained to the model with a matching number of true components. While not necessarily optimal nor theoretically justified, the benefit of this approach is no changes are required to the existing RJMCMC implementation. Also, it seemed to work quite well for the simulated data sets and the French mortality data where the empty components were observed.

Closing remarks

While our discussion mainly concerns the model posterior distribution for choosing the optimal number of mixture components, its use should not be limited. The topic, on the whole, is too broad for an in-depth review in the limits of this paper as, in a more general case, the interpretation of the model posterior distribution depends on the differences between the models in question.

We would also like to mention the approach of Guha et al. [2021]. They introduce the Merge-Truncate-Merge algorithm, which, together with some post-processing, supposedly solves the issue of false components for a mixture model. While their method can find the optimal number of components as is, their strategy could be used for merging any false components in the estimated Stan models before the RJMCMC step in the `CU-MSDSp`. This could be considered another strategy to obtain the rectified model posterior distribution.

3 Simulation study

This section contains results for the simulated data sets. We begin with an analysis of the TMixClust Toydata available with the R package `TMixClust` [Golumbeanu et al., 2019]. More information on creating the data set can be found in [Golumbeanu, 2020]. Here, we give a more in-depth outline of the process, followed by an analysis of our simulated data sets.

3.1 TMixClust Toydata

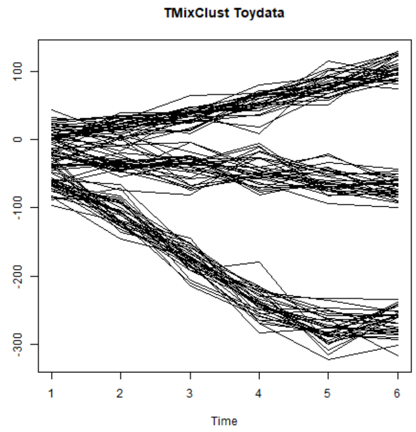

Figure 1: Plot of TMixClust toydata

Data preparation and the models

We begin by loading the simulated time-series data provided in `TMixClust` (Figure 1). Stan data object was created, which contains the data, smoothing prior and values for other fixed parameters. The object is then written into a JSON file (example function is found in the `R` file). As for the models, we estimated three nested models

$$\boldsymbol{X}|\boldsymbol{\theta} \sim \sum_{k=1}^{K} \pi_k f_k(\boldsymbol{X}|\boldsymbol{\theta}_k),$$

$K = 2, 3, 4$, with the hierarchical prior structure $f_k(\boldsymbol{X}|\boldsymbol{\theta}_k) = \mathcal{N}(\boldsymbol{X}|\boldsymbol{z}_k, \sigma^2)$, $z_{0,k} \sim \mathcal{N}(-30, 100)$, $z_{t,k} \sim \mathcal{N}(z_{t-1,k}, 15)$, $\sigma^2 \sim \Gamma^{-1}(1, 1)$, $\pi_k \sim \text{Dirichlet}(\mathbf{1})$. An order restriction is assumed on the prior distribution of the initial value of each random walk process to avoid label switching, a standard approach in mixture modelling through mean. For this example, we assume the same level of smoothness for each of the mixture components.

Model estimation

The posterior distributions of parameters of individual models are estimated through the `CU-MSDSp`; for details, see Appendix 6. We used different lengths of MCMC chains. Typically, the models converge by setting 'NBURN'= 4000. Additionally, 4000-15000 MCMC samples were collected after the warm-up.

Assignment

The model is fully probabilistic at this point, and none of the individual time series is labelled. With the assignment, we can use the parameter information to cluster the observations. We show our approach to this but note that many approaches to using the MCMC chain are at least partially subjective.

The first and easiest approach is to use built-in R functions to calculate the posterior medians for all the model parameters. This is because the median is a more robust estimator for the chain than the mean for possibly skewed posterior distributions. We plot the estimated cluster centres (Figure 2a). These are the means of the random walk processes. We note that these are not mean values for the observed data but random numbers that are likely behind the observed data. In our fitted models, these are denoted by l1.1, l1.2, and so on and are collected to L1, L2,. . . . These can be plotted on the data to get an idea of how the clusters will be located, but also, it is quite easy to spot if something goes wrong (bad model fit).

Next, we apply the assignment estimation. We use the posterior estimated mixture weights and variance parameters for our function. The main part of the function consists of an exponential function, the argument of which we can put the distance function of choice. In our case, the distance function is the Euclidean distance to a cluster's centre weighted by the mixture components' estimated variance parameter. Applying this function to the individual time

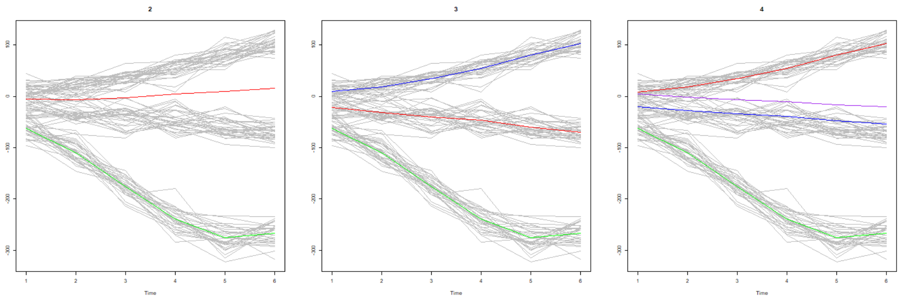

(a) Estimated centres of the mixture components for $K = 2, 3, 4$.

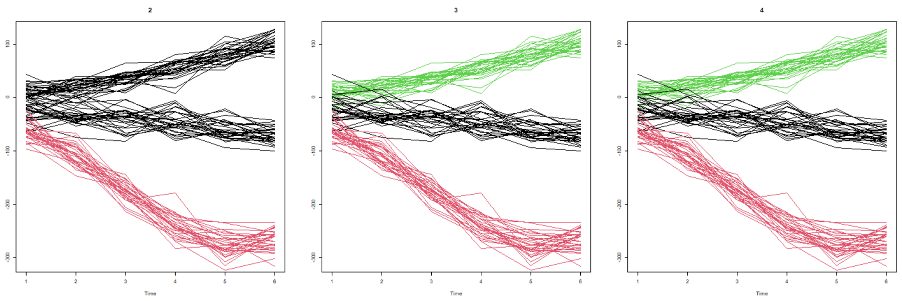

(b) Cluster assignments for $K = 2, 3, 4$.

Figure 2: Estimated centres of the mixture components plotted on the data and the resulting clusters given by the BELMM approach. Results from left to right for $K = 2, 3, 4$ respectively.

series, we get a matrix of clusters' unnormalised probabilities, i.e., a prototype cluster membership matrix. These are not necessarily probabilities, but since the normalisation does not change their order, they can be used for assigning the observation to the most plausible cluster. The size of the clusters can be checked with `table()` function and plotted with `ts.plot()` or other similar function (Figure 2b). If the resulting assignment is favourable, we can stop here.

Thresholding

We may apply thresholding if the clusters are too big or noisy to conclude. Applying built-in functions to the prototype matrix is easily done in R. With `ts.plot()`, one can use trial-and-error to set the threshold until the clusters are visible or of the desired size. Since the prototype probabilities are unnormalised, we cannot treat the membership thresholds as probabilities (or credible intervals for the clusters). The constants needed for probability interpretation are generally hard to calculate (cf. Bayes factor). However, it is still analogous to the classic credible interval. For each cluster we threshold, we also create a 'junk'

group of observations that are not strong members of the clusters. For extra information, we may iterate the RJMCMC to see if any interesting clusters are formed within the ‘noise’.

	K	π_1	π_2	π_3	π_4
Estimates	2	0.65666	0.34334		
	3	0.33003	0.33995	0.33002	
	4	0.32760	0.33570	0.21371	0.12299
Realised	2	0.65934	0.34066		
	3	0.32967	0.34066	0.32967	
	4	0.32967	0.34066	0.32967	0

Table 1: Summary of posterior estimated weights and realisations after the assignments given by BELMM approach, TMixClust Toydata

Analysis of the model posterior distribution

Next, we may compare the results from the individual models against the model posterior distribution. Since some components were left empty with nested models (Figure 2b), the probability could be transferred to form the rectified model posterior distribution (Figure 3).

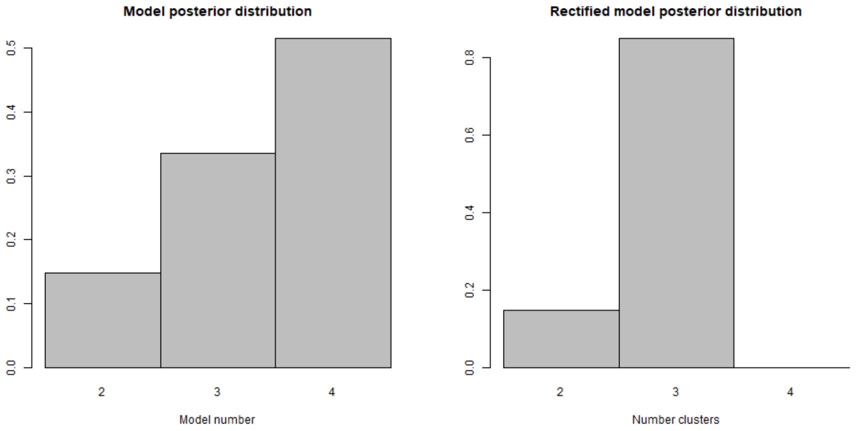

Figure 3: Original Model posterior distribution given by `CU-MSDSp` and the associated rectified model posterior distribution.

Comparison: TMixClust results

This subsection is about the results of applying TMixClust to the Toydata. Clusters found by TMixClust for $K = 2, 3, 4$ are shown in Figure 4, and the

estimated mixture weights in Table 2.

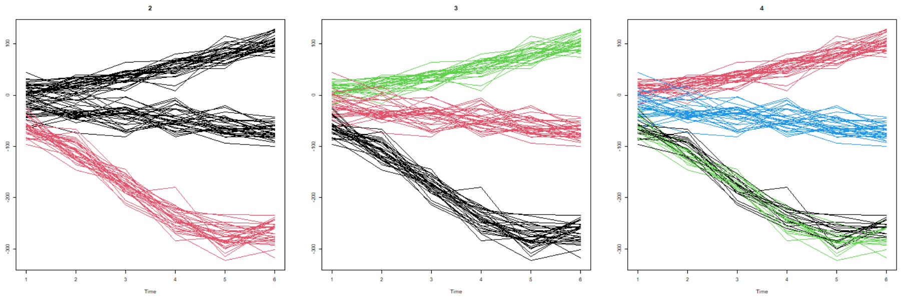

Figure 4: TMixClust clusters.

	K	π_1	π_2	π_3	π_4
Estimates	2	0.65934	0.34066		
	3	0.34066	0.32967	0.32967	
	4	0.16484	0.32967	0.17582	0.32967

Table 2: Summary of estimates given by applying TMixClust method.

3.1.1 Comparison of smoothing distributions

Comparison of Random walk distributions

This short section is about the choice of the smoothing distribution. The model is

$$\boldsymbol{X}|\boldsymbol{\theta} \sim \sum_{k=1}^{K} \pi_k f_k(\boldsymbol{X}|\boldsymbol{\theta}_k).$$

Here, we assume each mixture component is normally distributed, but the underlying random walk $\boldsymbol{z}_k$ is either Cauchy, Laplace, Normal, or Student's t distributed. The model in terms of hierarchical priors is $f_k(\boldsymbol{X}|\boldsymbol{\theta}_k) = \mathcal{N}(\boldsymbol{X}|\boldsymbol{z}_k, \sigma^2)$, $z_{0,k} \sim \psi(-30, 20)$, $z_{t,k} \sim \psi(z_{t-1,k}, 15)$, $\sigma^2 \sim \Gamma^{-1}(1, 1)$, $\pi_k \sim$ Dirichlet($\mathbf{1}$), where ψ is Cauchy, Laplace, Normal, or Student's t distribution. The estimated centres and the associated model posterior distribution are shown in Figure 5.

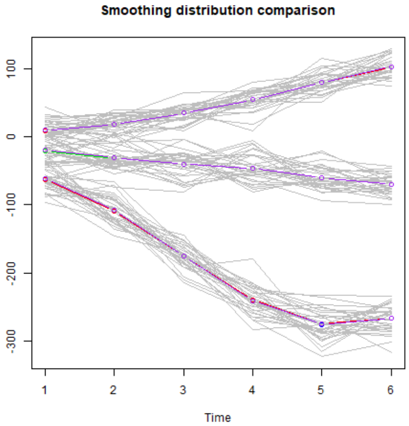

(a) Estimated centres.

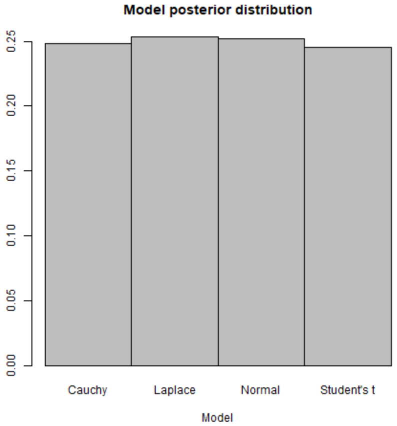

(b) Model posterior distribution given by `CU-MSDSp`.

Figure 5: Comparison of the random walk smoothing distributions: Cauchy, Laplace, Normal, and Student's t.

Mixture distributions comparison

In the following, we assume the latent processes follow AR(1)-based centres. The mixture components are assumed to be either Cauchy, Laplace, Normal, or Student's t distributed.

The model is

$$\boldsymbol{X}|\boldsymbol{\theta} \sim \sum_{k=1}^{K} \pi_k f_k(\boldsymbol{X}|\boldsymbol{\theta}_k).$$

Here, we assume each mixture component is either Cauchy, Laplace, Normal, or Student's t distributed, but the underlying random walk $\boldsymbol{z}_k$ follows an AR(1) process. The model in terms of hierarchical priors is $f_k(\boldsymbol{X}|\boldsymbol{\theta}_k) = \psi(\boldsymbol{X}|\boldsymbol{z}_k, \sigma^2)$, $z_{0,k} \sim \psi\left(-30, 20\right)$, $z_{t,k} \sim \psi\left(z_{t-1,k}, 15\right)$, $\sigma^2 \sim \Gamma^{-1}\left(1, 1\right)$, $\pi_k \sim \text{Dirichlet}(\mathbf{1})$, where ψ is Cauchy, Laplace, Normal, or Student's t distribution. The degrees of freedom value we used was $T - 1 = 5$. The estimated centres and the associated model posterior distribution are shown in Figure 6a, 6b, respectively.

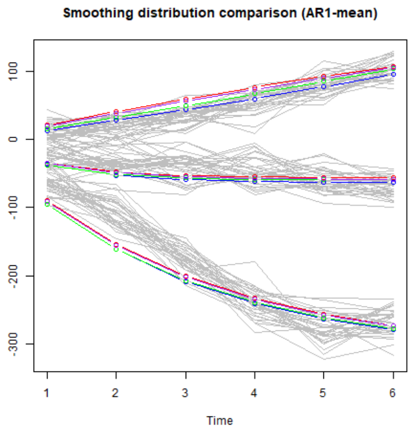

(a) Estimated centres.

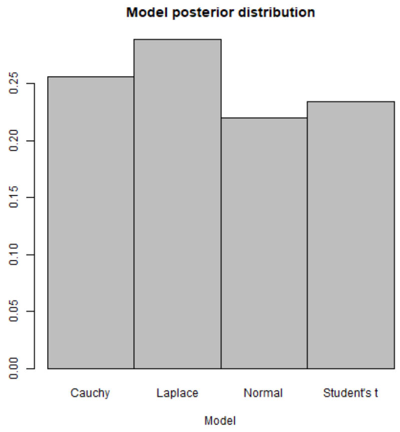

(b) Model posterior distribution given by `CU-MSDSp`.

Figure 6: Comparison of mixture distributions with AR(1)-based mean: Cauchy, Laplace, Normal, Student's t.

3.2 Simulated data sets

In this subsection, we show the results for the five simulated time series data sets. The data sets are simulated by drawing 100 series according to the mixture distribution

$$\boldsymbol{X}|\boldsymbol{\theta} \sim \sum_{k=1}^{K} \pi_k f_k(\boldsymbol{X}|\boldsymbol{\theta}_k),$$

where for $K = 1, \ldots, 5$ the mixture distributions f_i are

$$f_1(x) \sim \mathcal{N}\left(2 \cdot \sin(\mathcal{N}\left(x, 0.25^2\right)), 0.25^2\right)$$

$$f_2(x) \sim \mathcal{N}\left(2 \cdot \cos(\mathcal{N}\left(x, 0.25^2\right)), 0.5^2\right)$$

$$f_3(x) \sim \mathcal{N}\left(\sqrt{3 + \mathcal{N}\left(2 \cdot x, 0.1^2\right)}, 0.5^2\right)$$

$$f_4(x) \sim \mathcal{N}\left(-0.5 - x \cdot \sin(x), 0.25^2\right)$$

$$f_5(x) \sim \mathcal{N}\left(8, 0.5^2\right),$$

where $x = (0, 0.1, 0.2, \ldots, 6.2)$. The following mixture weights were used in the simulation: For single-component data, $\pi_1 = 1$. For two-component data, $\pi_1 = 0.33, \pi_2 = 0.67$. For three-component data, $\pi_1 = 0.33, \pi_2 = 0.33, \pi_3 = 0.34$. For four-component data, $\pi_1 = 0.33, \pi_2 = 0.33, \pi_3 = 0.19, \pi_4 = 0.15$. For five-component data, $\pi_1 = 0.33, \pi_2 = 0.33, \pi_3 = 0.19, \pi_4 = 0.1, \pi_5 = 0.05$.

3.2.1 Single component

The simulated data is shown in Figure 7, the model posterior distribution and the rectified model posterior distribution in 8. The estimated mixture component centres and the resulting clusters are shown in 9a and 9b, respectively. The summary of the results is given in Table 3.

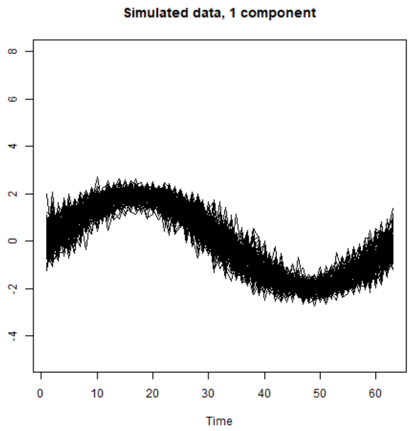

Figure 7: Plot of individual time series from the simulated single-component data.

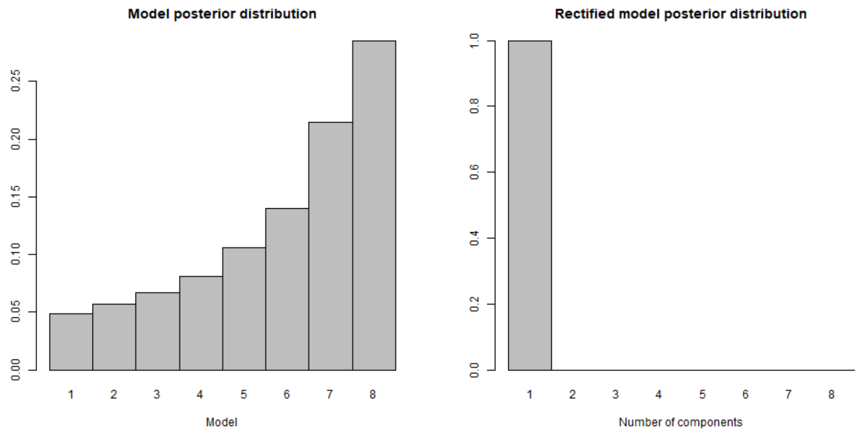

Figure 8: Original Model posterior distribution given by CU-MSDSp and the associated rectified model posterior distribution: Estimated from the single-component data.

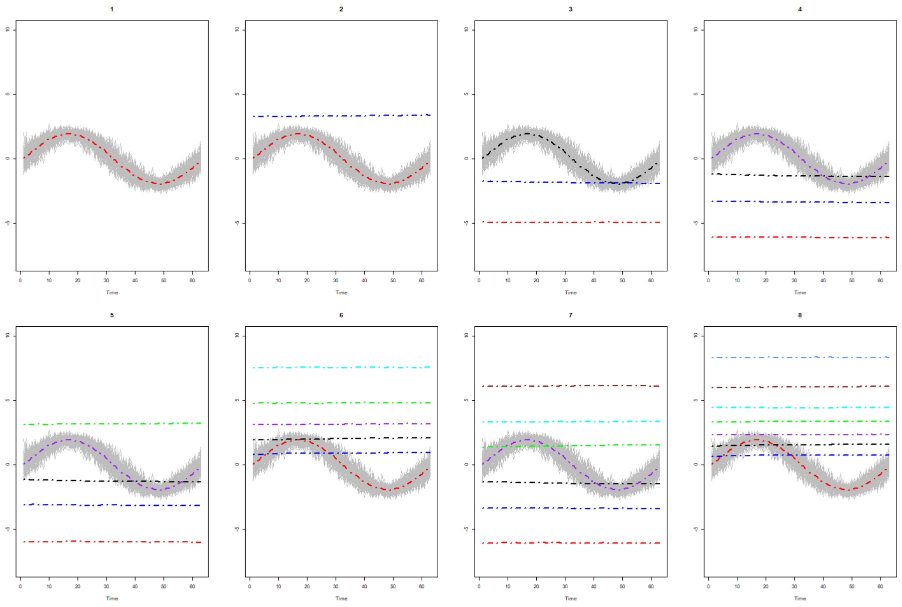

(a) Estimated centres.

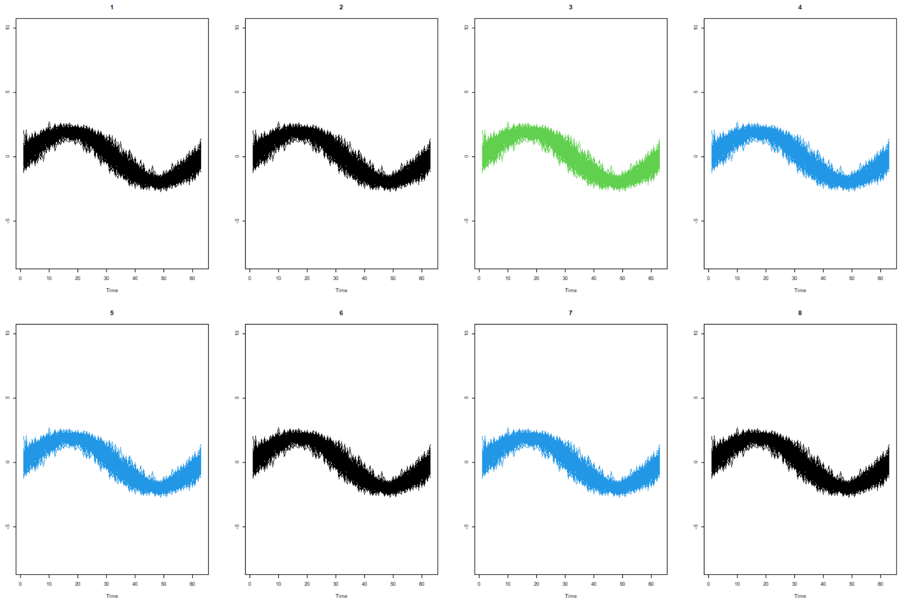

(b) Clusters.

Figure 9: Estimated centres and cluster assignments given by the BELMM approach for the single-component data.

	K	π_1	π_2	π_3	π_4	π_5	π_6	π_7	π_8
Estimates	1	1							
	2	0.99323	0.00677						
	3	0.00666	0.00668	0.98355					
	4	0.00664	0.00686	0.00679	0.97414				
	5	0.00688	0.00653	0.00665	0.96451	0.00674			
	6	0.95521	0.00652	0.00664	0.00664	0.00662	0.00644		
	7	0.00648	0.00655	0.00650	0.94677	0.00649	0.00645	0.00650	
	8	0.93805	0.00659	0.00632	0.00636	0.00652	0.00648	0.00644	0.00650
Realised	1	1							
	2	1	0						
	3	0	0	1					
	4	0	0	0	1				
	5	0	0	0	1	0			
	6	1	0	0	0	0	0		
	7	0	0	0	1	0	0	0	
	8	1	0	0	0	0	0	0	0

Table 3: Summary of the estimated and realised values of the mixture weights after assignment given by the BELMM approach for the single-component data.

3.2.2 Two components

The simulated data is shown in Figure 10, the model posterior distribution and the rectified model posterior distribution in 11. The estimated mixture component centres and the resulting clusters are shown in 12a and 12b, respectively. The summary of the results is given in Table 4.

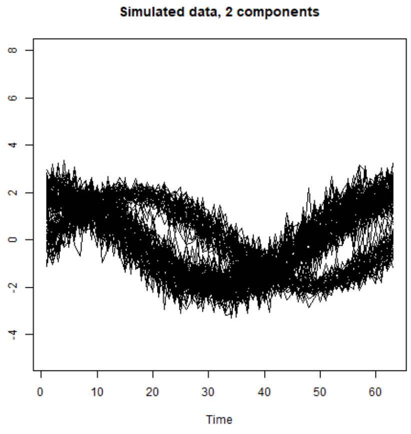

Figure 10: Plot of individual time series from the simulated two-component data.

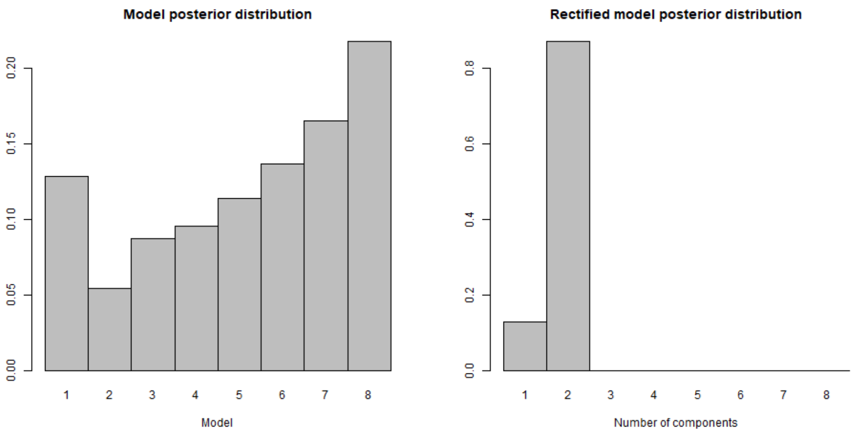

Figure 11: Original Model posterior distribution given by `CU-MSDSp` and the associated rectified model posterior distribution: Estimated from the two-component data.

	K	π_1	π_2	π_3	π_4	π_5	π_6	π_7	π_8
Estimates	1	1							
	2	0.62889	0.37111						
	3	0.36823	0.00677	0.62231					
	4	0.00679	0.00672	0.61629	0.36443				
	5	0.00664	0.00672	0.61018	0.36157	0.00668			
	6	0.00654	0.00652	0.60523	0.35700	0.00663	0.00647		
	7	0.00632	0.00646	0.59852	0.35465	0.00654	0.00655	0.00655	
	8	0.00635	0.00641	0.59384	0.35080	0.00644	0.00632	0.00640	0.00650
Realised	1	1							
	2	0.63	0.37						
	3	0	0	0.63					
	4	0	0	0.63	0.37				
	5	0	0	0.63	0.37	0			
	6	0	0	0.63	0.37	0	0		
	7	0	0	0.63	0.37	0	0	0	
	8	0	0	0.63	0.37	0	0	0	0

Table 4: Summary of the estimated and realised values of the mixture weights after assignment given by the BELMM approach for the two-component data.

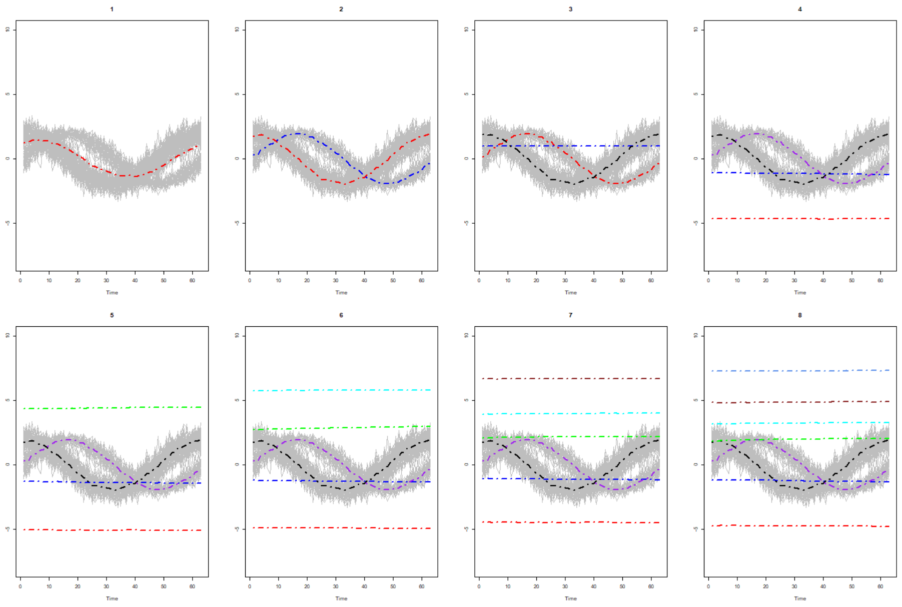

(a) Estimated centres.

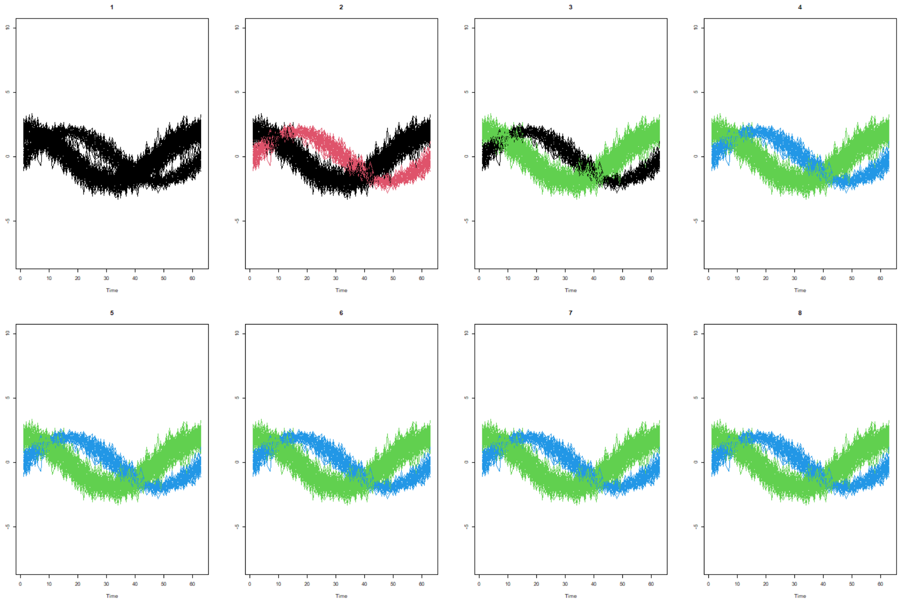

(b) Clusters.

Figure 12: Estimated centres and cluster assignments given by BELMM approach for the two-component data.

3.2.3 Three components

The simulated data is shown in Figure 13, the model posterior distribution and the rectified model posterior distribution in 14. The estimated mixture component centres and the resulting clusters are shown in 15a and 15b, respectively. The summary of the results is given in Table 5.

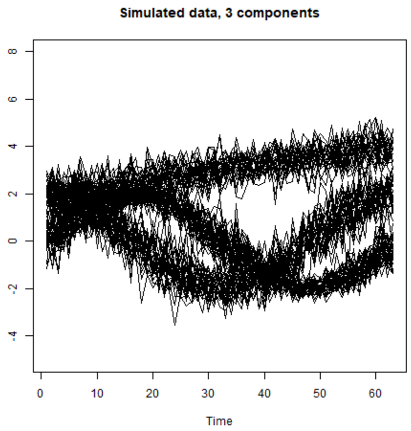

Figure 13: Plot of individual time series from the simulated three-component data.

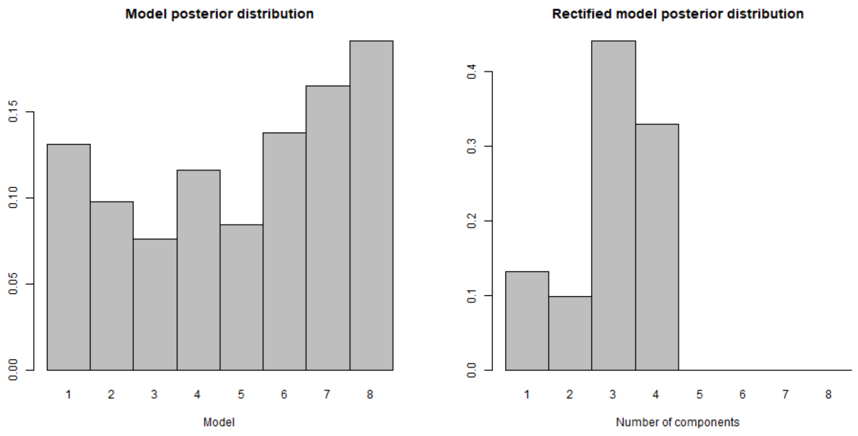

Figure 14: Original Model posterior distribution given by CU-MSDSp and the associated rectified model posterior distribution: Estimated from the three-component data.

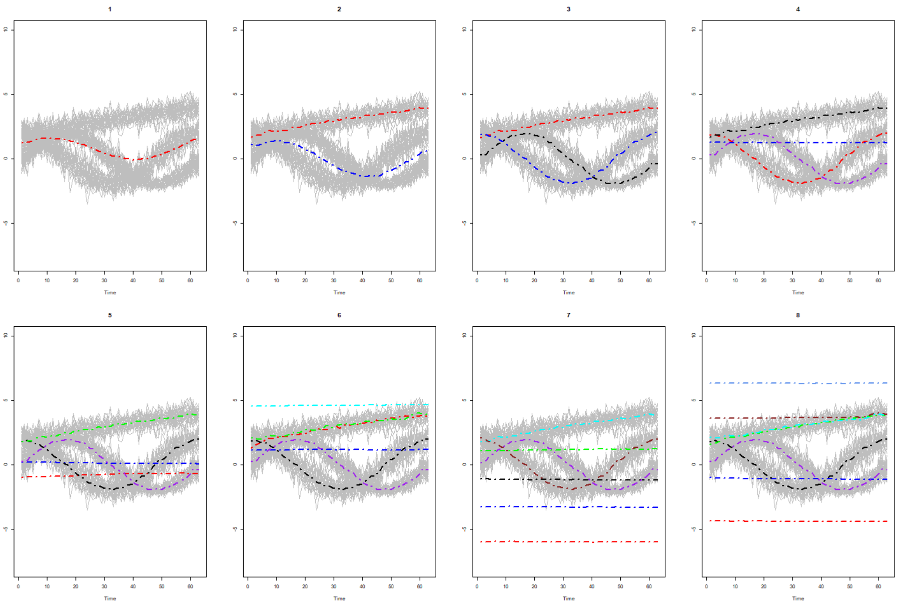

(a) Estimated centres.

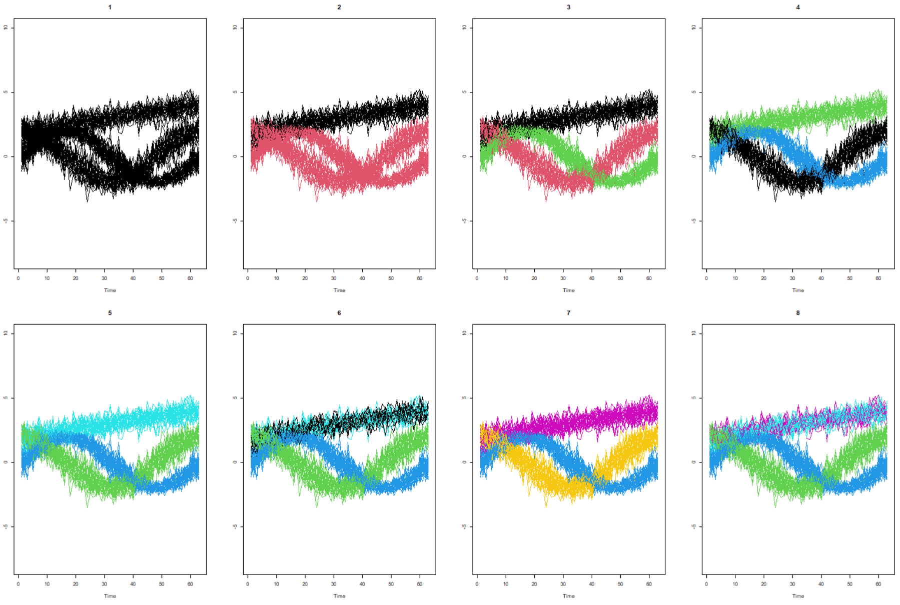

(b) Clusters.

Figure 15: Estimated centres and cluster assignments given by BELMM approach for the three-component data.

	K	π_1	π_2	π_3	π_4	π_5	π_6	π_7	π_8
Estimates	1	1							
	2	0.28264	0.71736						
	3	0.28060	0.30928	0.40686					
	4	0.30683	0.00683	0.27606	0.40360				
	5	0.00929	0.00668	0.30328	0.39988	0.26325			
	6	0.13207	0.00814	0.30073	0.39618	0.13566	0.00680		
	7	0.00658	0.00623	0.00664	0.39147	0.00793	0.26208	0.29794	
	8	0.00657	0.00650	0.29488	0.38931	0.18099	0.07635	0.00717	0.00668
Realised	1	1							
	2	0.28	0.72						
	3	0.28	0.31	0.41					
	4	0.31	0	0.28	0.41				
	5	0	0	0.31	0.41	0.28			
	6	0.15	0	0.31	0.41	0.13	0		
	7	0	0	0	0.41	0	0.28	0.31	
	8	0	0	0.31	0.41	0.16	0.12	0	0

Table 5: Summary of the estimated and realised values of the mixture weights after assignment given by the BELMM approach for the three-component data.

3.2.4 Four components

The simulated data is shown in Figure 16, the model posterior distribution and the rectified model posterior distribution in 17. The estimated mixture component centres and the resulting clusters are shown in 18a and 18b, respectively. The summary of the results is given in Table 6.

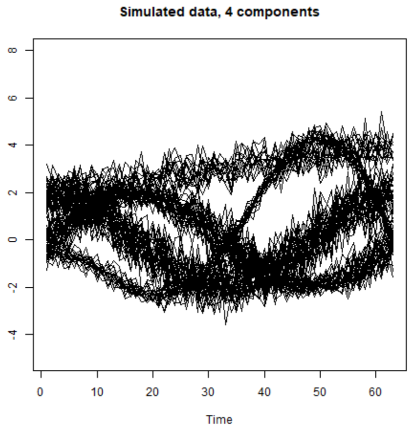

Figure 16: Plot of individual time series from the simulated four-component data.

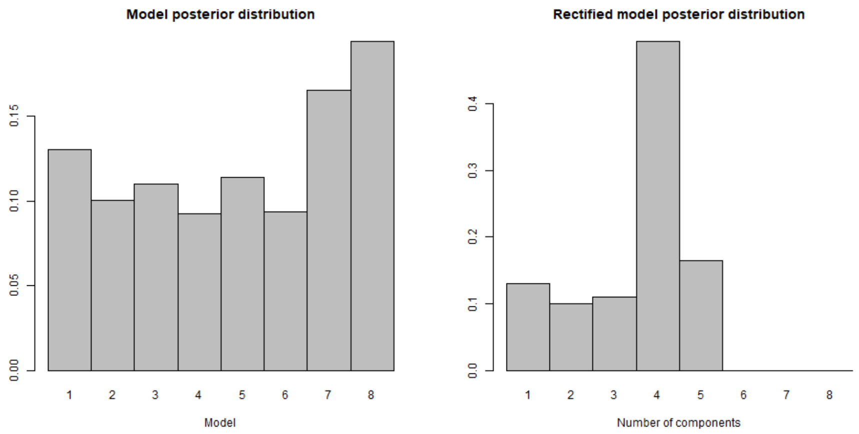

Figure 17: Original Model posterior distribution given by CU-MSDSp and the associated rectified model posterior distribution: Estimated from the four-component data.

	K	π_1	π_2	π_3	π_4	π_5	π_6	π_7	π_8
Estimates	1	1							
	2	0.31316	0.68684						
	3	0.30992	0.34935	0.33774					
	4	0.17060	0.14219	0.33595	0.34497				
	5	0.16884	0.00668	0.33240	0.34115	0.14147			
	6	0.16800	0.00662	0.00671	0.32874	0.33946	0.13948		
	7	0.16597	0.32496	0.33547	0.13798	0.00656	0.00655	0.00670	
	8	0.00647	0.00646	0.16435	0.32318	0.00661	0.00660	0.33256	0.13693
Realised	1	1							
	2	0.31	0.69						
	3	0.31	0.35	0.34					
	4	0.17	0.14	0.34	0.35				
	5	0.17	0	0.34	0.35	0.14			
	6	0.17	0	0	0.34	0.35	0.14		
	7	0.17	0.34	0.35	0.13	0.01	0	0	
	8	0	0	0.17	0.34	0	0	0.35	0.14

Table 6: Summary of the estimated and realised values of the mixture weights after assignment given by the BELMM approach for the four-component data.

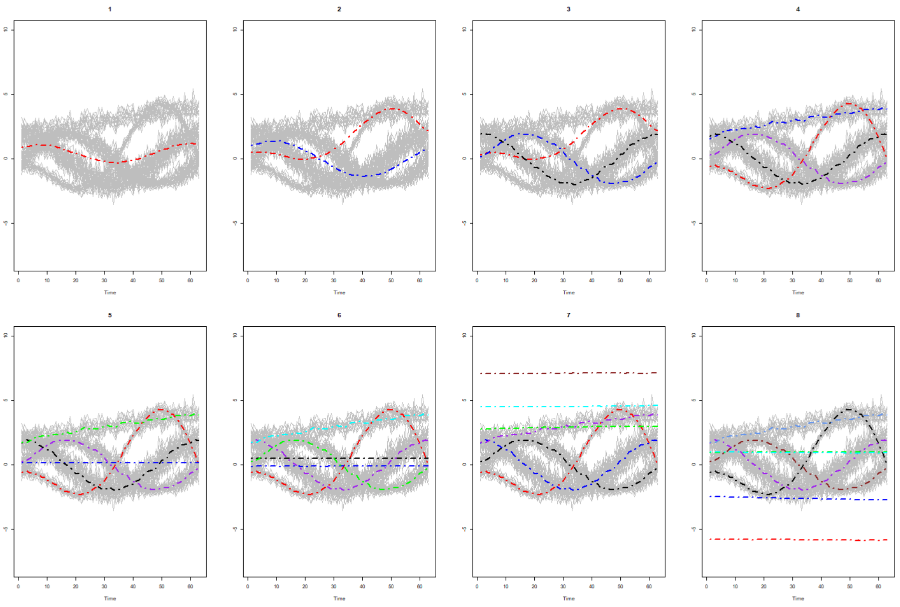

(a) Estimated centres.

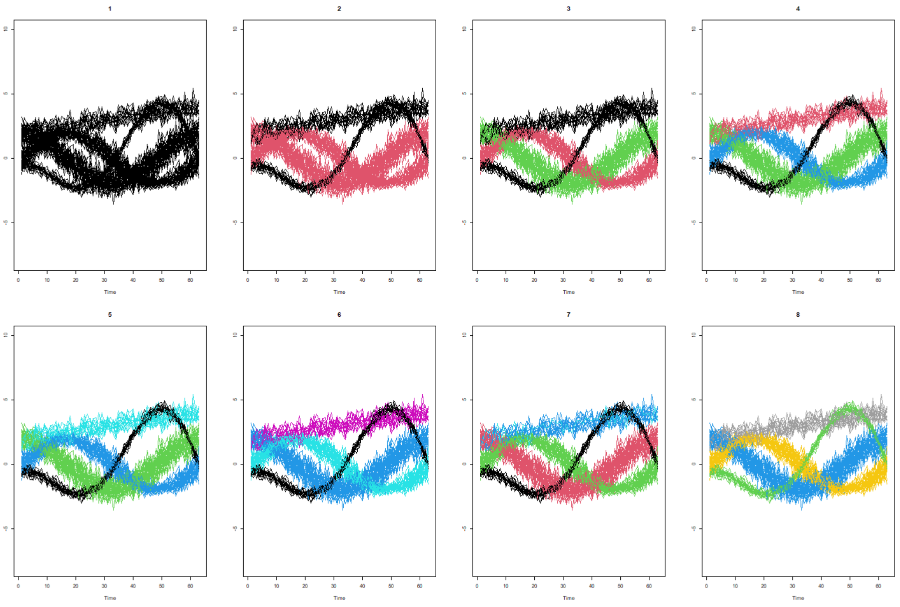

(b) Clusters.

Figure 18: Estimated centres and cluster assignments given by the BELMM approach for the four-component data.

3.2.5 Five components

The simulated data is shown in Figure 19, the model posterior distribution and the rectified model posterior distribution in 20. The estimated mixture component centres and the resulting clusters are shown in 21a and 21b, respectively. The summary of the results is given in Table 7.

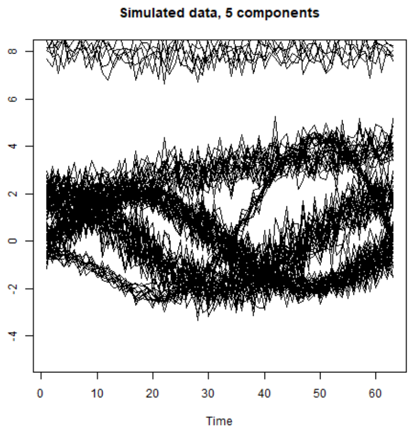

Figure 19: Plot of individual time series from the simulated five-component data.

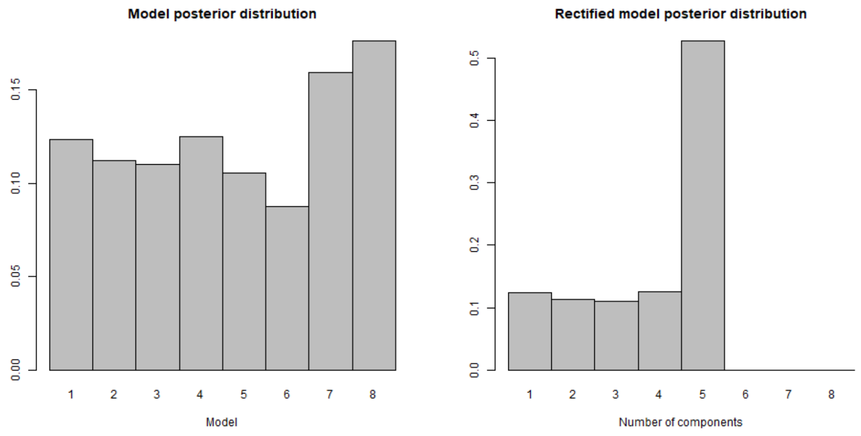

Figure 20: Original Model posterior distribution given by `CU-MSDSp` and the associated rectified model posterior distribution: Estimated from the five-component data.

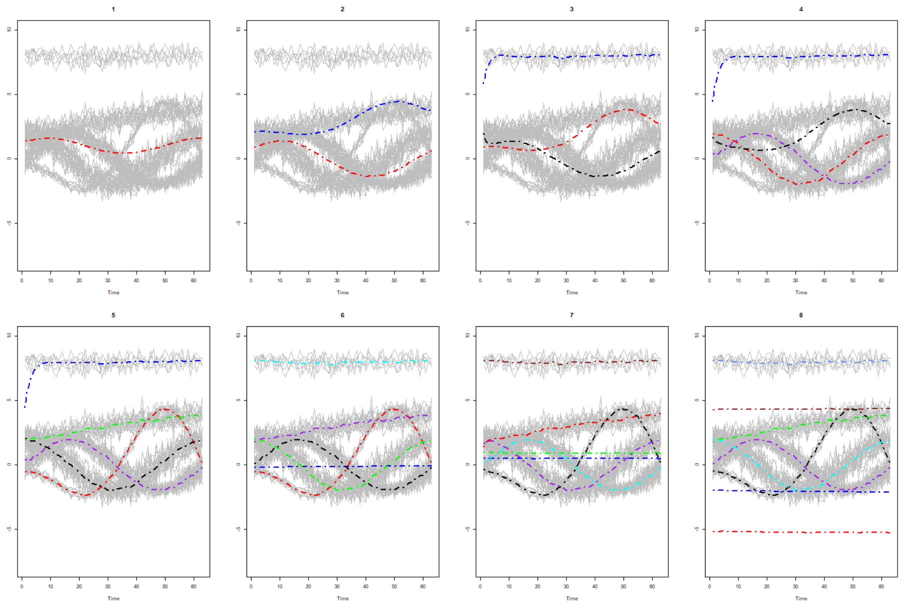

(a) Estimated centres.

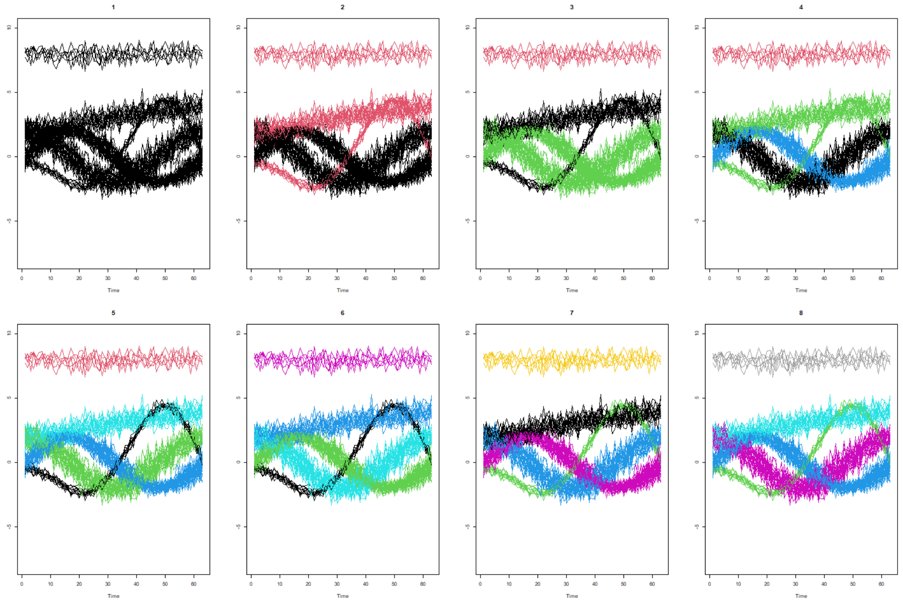

(b) Clusters.

Figure 21: Estimated centres and cluster assignments given by BELMM approach for the five-component data.

	K	π_1	π_2	π_3	π_4	π_5	π_6	π_7	π_8
Estimates	1	1							
	2	0.63795	0.36205						
	3	0.29871	0.06462	0.63352					
	4	0.26760	0.06495	0.29681	0.36490				
	5	0.12174	0.06411	0.26576	0.36124	0.17837			
	6	0.11986	0.00656	0.35835	0.17727	0.26258	0.06330		
	7	0.17491	0.00658	0.11862	0.26038	0.00657	0.35465	0.06323	
	8	0.00657	0.00655	0.11810	0.35077	0.17404	0.25790	0.00659	0.06232
Realised	1	1							
	2	0.64	0.36						
	3	0.30	0.06	0.64					
	4	0.27	0.06	0.30	0.37				
	5	0.12	0.06	0.27	0.37	0.18			
	6	0.12	0	0.37	0.18	0.27	0.06		
	7	0.18	0	0.12	0.27	0	0.37	0.06	
	8	0	0	0.12	0.37	0.18	0.27	0	0.06

Table 7: Summary of the estimated and realised values of the mixture weights after assignment given by the BELMM approach for the five-component data.

4 French mortality

This section summarises the results from empirical analysis 3, the French mortality. The section is structured as follows: First, we consider Interpretation 1 of the data. We provide the results for the RJMCMC, an example Stan model, and the estimates for the mixture components and the assigned clusters, respectively. We give an example of the rectified model posterior distribution based on these. We show results for the slow versions of the Stan model (see Subsection 6.3). The results from package `TMixClust` accompany the above. Second, the above results are provided for Interpretation 2 of the data.

The model is

$$\boldsymbol{X}|\boldsymbol{\theta} \sim \sum_{k=1}^{K} \pi_k f_k(\boldsymbol{X}|\boldsymbol{\theta}_k).$$

Here, we assume each mixture component is normally distributed, and the underlying random walk $\boldsymbol{z}_k$ is normally distributed. The model in terms of hierarchical priors is $f_k(\boldsymbol{X}|\boldsymbol{\theta}_k) = \mathcal{N}(\boldsymbol{X}|\boldsymbol{z}_k, \sigma^2)$, $z_{0,k} \sim \mathcal{N}(-3, 6)$, $z_{t,k} \sim \mathcal{N}(z_{t-1,k}, 0.15)$, $\sigma^2 \sim \Gamma^{-1}(1, 1)$, $\pi_k \sim \text{Dirichlet}(\mathbf{1})$.

4.1 Interpretation 1: 203 profiles of length 101

The French mortality data and the model posterior distribution are shown in Figure 22a and 22b, respectively. The rectified model posterior distribution is in 25. The estimated mixture component centres and the resulting clusters for the seven component model are shown in 24a and 24b, respectively. The summary of the results is given in Table 8.

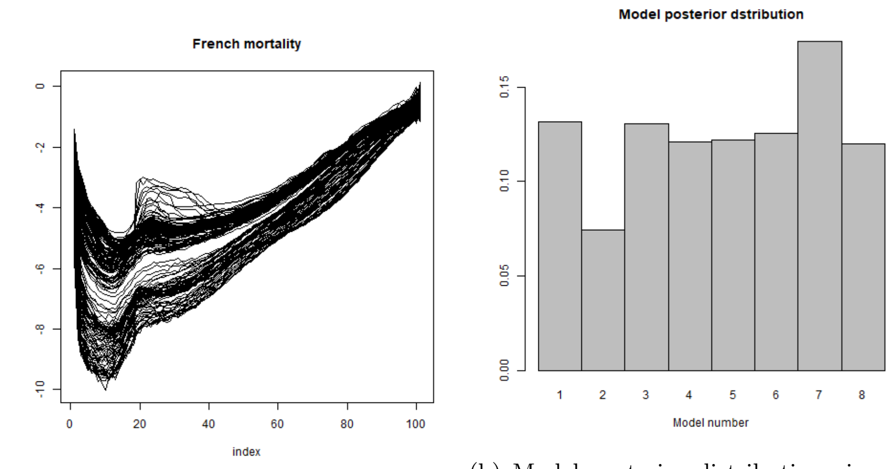

(a) Data

(b) Model posterior distribution given by `CU-MSDSp`

Figure 22: Log-transformed French mortality data and the model posterior distribution for the slow implementation.

For comparison, a different number of models affects the model posterior distribution. Figure 23 shows the model posterior distribution for the first five models. On the left is the model posterior distribution from the last 4000 RJMCMC samples. On the right is the model posterior distribution from the full 8000 samples RJMCMC chain. We see that the chain has converged fast. The estimated centres are shown in Figure 26. A summary of the results for the fast implementation is given in Table 9.

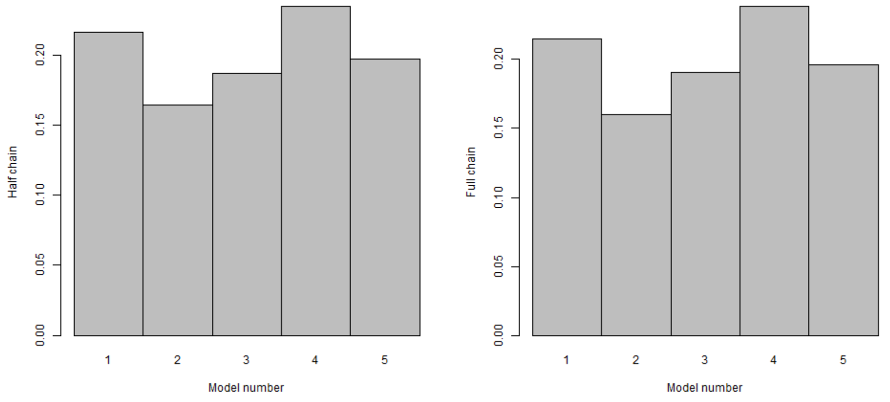

Figure 23: The French mortality data: Model posterior distribution for five models with the slow implementation.

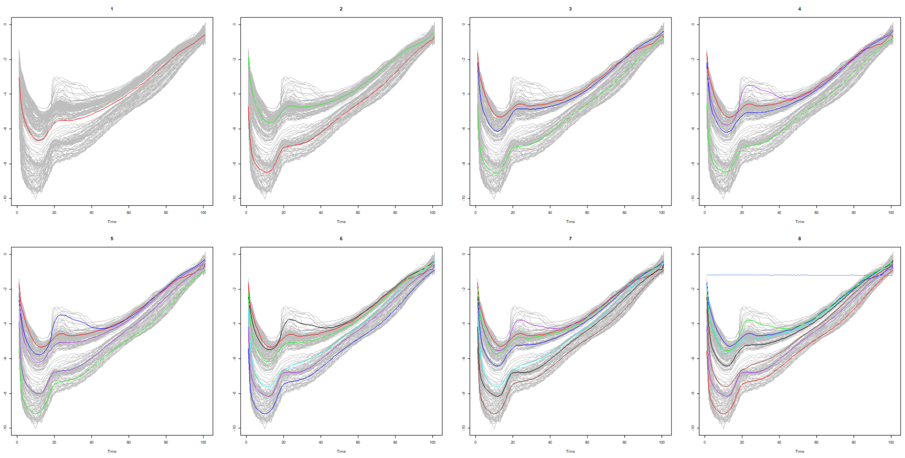

(a) Centres

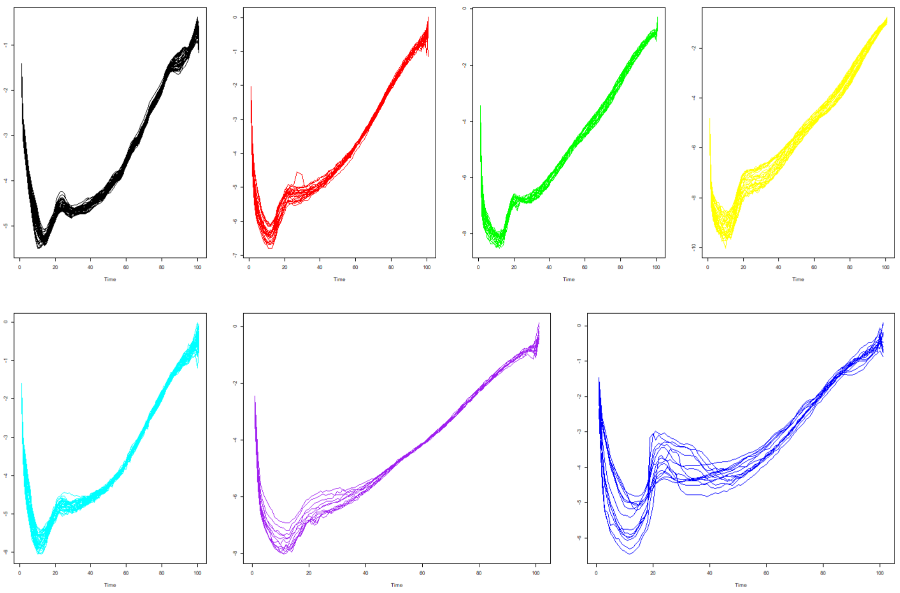

(b) Clusters after assignment.

Figure 24: Estimates of the mixture components' centres for eight models with the slow implementation given by BELMM. Centres are on the top plot. The realised clusters for the seven-component model are on the bottom plot.

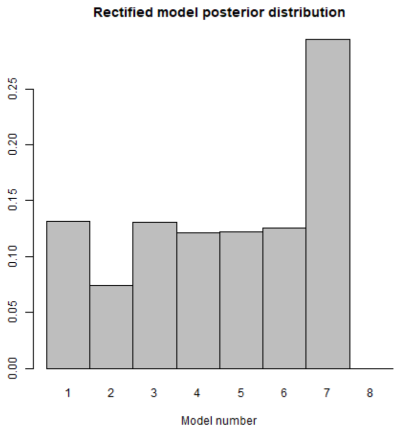

Figure 25: The rectified model posterior distribution for the slow implementation

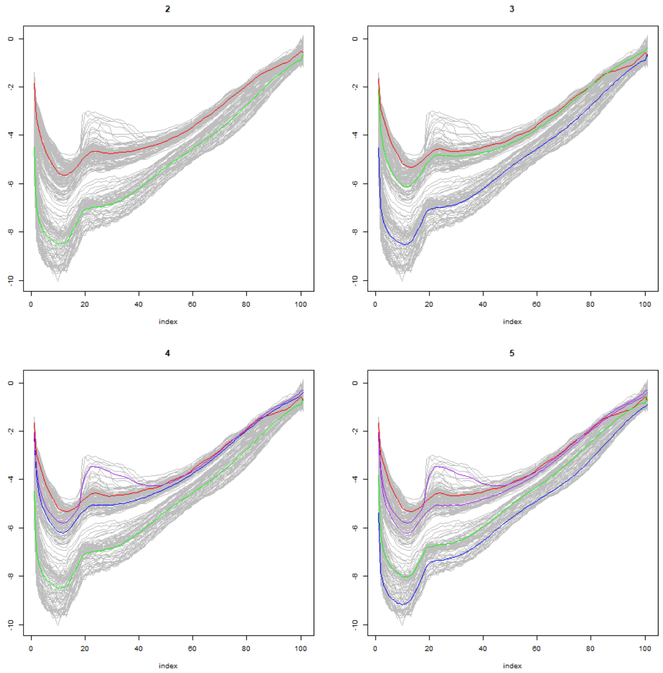

Figure 26: Estimated centres for models 2-5 with the fast implementation given by BELMM.

	K	π_1	π_2	π_3	π_4	π_5	π_6	π_7	π_8
Estimates	7	0.24266	0.12708	0.14106	0.06999	0.19307	0.06016	0.15571	-
Realised	7	0.24631	0.12808	0.14286	0.06897	0.19704	0.05911	0.15764	-
Estimates	8	0.15493	0.24225	0.12681	0.14076	0.06972	0.19263	0.06047	0.00318
Realised	8	0.15764	0.24631	0.12808	0.14286	0.06897	0.19704	0.05911	0

Table 8: Summary of the seven and eight component results given by BELMM, French mortality, slow implementation.

	K	π_1	π_2	π_3	π_4	π_5
Estimates	2	0.63936	0.36064			
	3	0.36140	0.28189	0.35422		
	4	0.37640	0.35678	0.22076	0.04200	
	5	0.37472	0.19621	0.15761	0.22494	0.04166
Realised	2	0.64039	0.35961			
	3	0.35468	0.29064	0.35468		
	4	0.37931	0.35468	0.22660	0.03941	
	5	0.37931	0.19704	0.15764	0.22660	0.03941

Table 9: Summary of results for models 2-5 given by BELMM, French mortality, fast implementation.

Comparison: TMixClust

To compare the clustering solutions and the estimated weights obtained with the BELMM approach, we provide some results for the package `TMixClust`. Cluster results for 2-5 clusters are shown in Figure 27 and the summary of the results is given in Table 10.

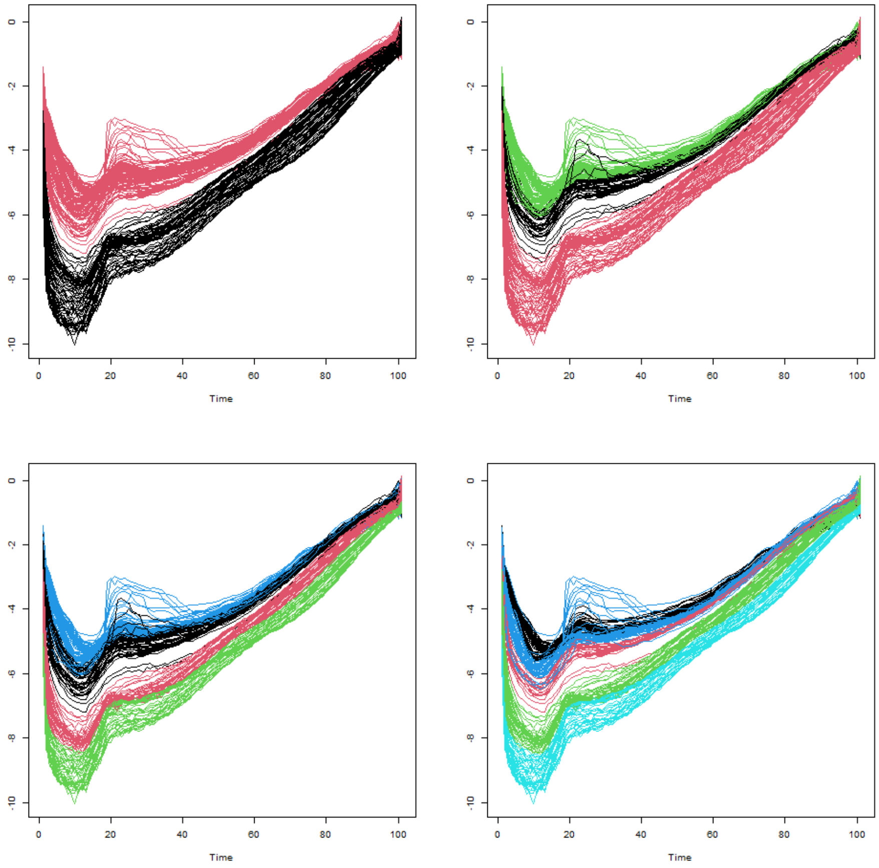

Figure 27: Results given by TMixClust method for the French mortality data.

	K	π_1	π_2	π_3	π_4	π_5
Estimates	2	0.34975	0.65025			
	3	0.19704	0.34483	0.45813		
	4	0.22167	0.18227	0.16749	0.42857	
	5	0.32020	0.11330	0.19212	0.21675	0.15764

Table 10: Summary of the results given by TMixClust method, French mortality data.

4.2 Interpretation 2: 101 time series of length 203

The French mortality data and the model posterior distribution are shown in Figure 28a and 28b, respectively. The realised clusters for all eight models and

the resulting clusters for the four and eight-component models are shown in 29a and 29b, respectively. The summary of the results is given in Table 11, and a summary for 5 models with fast implementation is given in Table 12 for comparison.

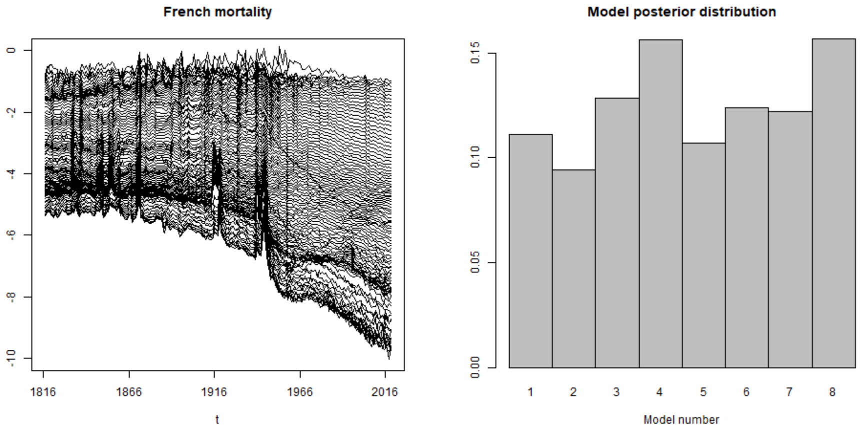

(a) The French mortality data.

(b) Model posterior distribution

Figure 28: The French mortality, Interpretation 2. The data set and the model posterior distribution.

	K	π_1	π_2	π_3	π_4	π_5	π_6	π_7	π_8
Estimates	2	0.44029	0.55971						
	3	0.22828	0.44933	0.32243					
	4	0.18925	0.37541	0.22467	0.21082				
	5	0.18521	0.18031	0.20859	0.17583	0.25001			
	6	0.14633	0.18761	0.15720	0.18195	0.18890	0.13801		
	7	0.15495	0.06799	0.15459	0.14232	0.18244	0.17282	0.12484	
	8	0.12389	0.18407	0.16652	0.12589	0.05841	0.14306	0.09239	0.10574
Realised	2	0.58416	0.41584						
	3	0.29703	0.47525	0.22772					
	4	0.40594	0.18812	0.20792	0.19802				
	5	0.18812	0.39604	0.10891	0.17822	0.12871			
	6	0.20792	0.11881	0.18812	0.18812	0.11881	0.17822		
	7	0.13861	0.10891	0.15842	0.12871	0.08911	0.17822	0.19802	
	8	0.11881	0.15842	0.11881	0.11992	0.06925	0.14209	0.08608	0.11182

Table 11: The French mortality, Interpretation 2. Summary of the results for the eight models given by the BELMM approach.

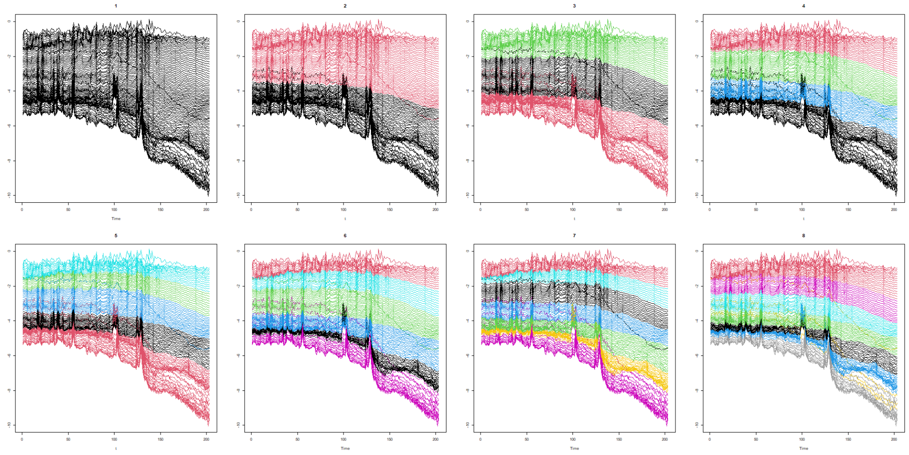

(a) Clusters, all models.

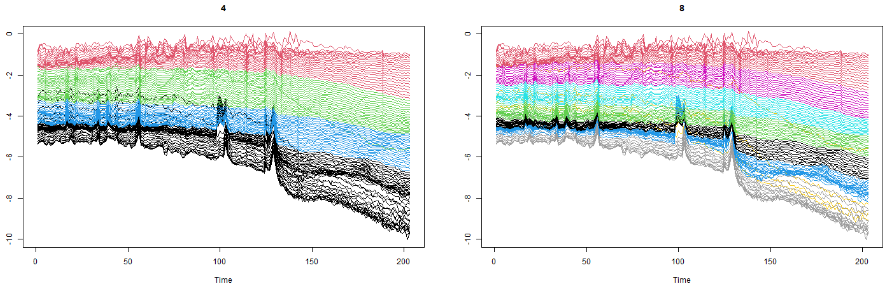

(b) Clusters for 4 and 8 models.

Figure 29: The French mortality, Interpretation 2. Estimated centres and assigned clusters given by BELMM approach.

	K	π_1	π_2	π_3	π_4	π_5
Estimates	2	0.58301	0.41699			
	3	0.29649	0.46150	0.23852		
	4	0.19779	0.39977	0.18797	0.20754	
	5	0.12015	0.16785	0.38455	0.17896	0.13940
Realised	2	0.58416	0.41584			
	3	0.29703	0.46535	0.23762		
	4	0.19802	0.40594	0.18812	0.20792	
	5	0.11881	0.16832	0.39604	0.17822	0.13861

Table 12: The French mortality, Interpretation 2. Summary of the results given by fast implementation of the BELMM approach.

Comparison: TMixClust

To compare the clustering solutions and the estimated weights obtained with the BELMM approach, we provide some results for the package `TMixClust`. Cluster results for 2-5 clusters are shown in Figure 30, and the summary of the results is given in Table 13.

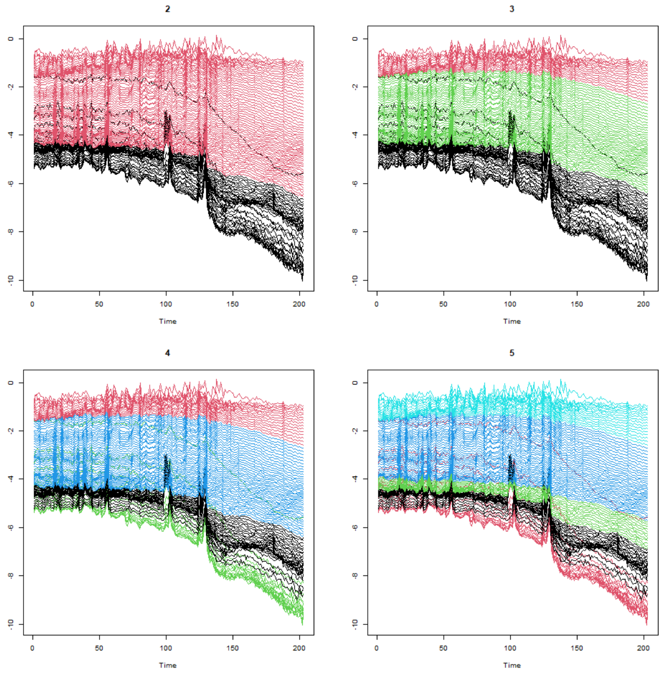

Figure 30: TMixClust results for The French mortality data, Interpretation 2.

	K	π_1	π_2	π_3	π_4	π_5
Estimates	2	0.42574	0.57426			
	3	0.44554	0.14851	0.40594		
	4	0.29703	0.14851	0.14851	0.40594	
	5	0.24752	0.14851	0.11881	0.33663	0.14851

Table 13: TMixClust results for The French mortality data, Interpretation 2, summary.

5 *Drosophila melanogaster* embryogenesis time-series data set

5.1 Analysis on full data

The *Drosophila melanogaster* data is shown in Figure 31. The silhouette index given by NbClust using the noted methods guiding the decision of the prior range and the model posterior distribution are shown in Figure 32a and 32b, respectively. The estimated mixture component centres and the resulting clusters for the eight models are shown in 33a and 33b, respectively. The summary of the results is given in Table 14.

For comparison, the estimated mixture component centres and resulting clusters are given for the 2-5 models estimated with the fast implementation in Figure 34a and 34b, respectively. Also, the summary for the 2-5 models is given in Table 15. The thresholded clusters and the rest of the genes are plotted in Figure 35a and 35b, and the summary of the sizes with different values for threshold are given in Table 16.

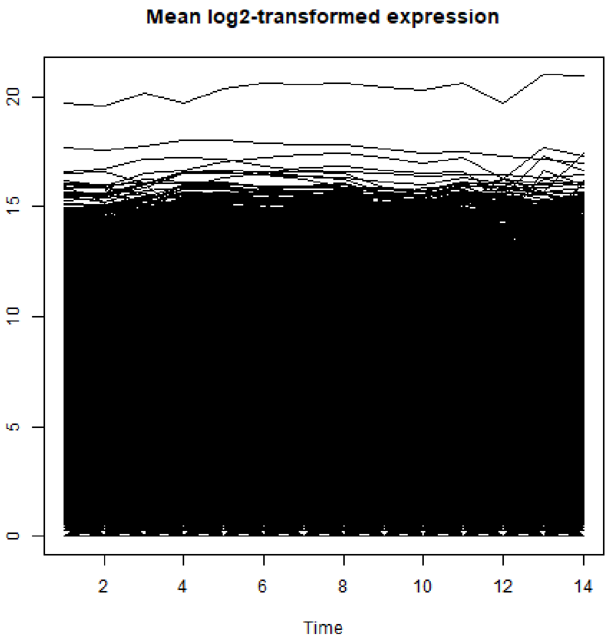

Figure 31: Data set.

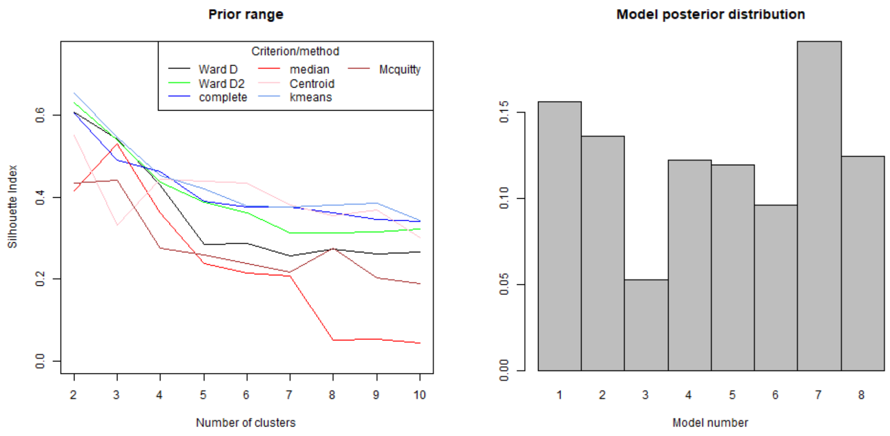

(a) Prior range. (b) Model posterior distribution

Figure 32: Prior range for the number of components given by NbClust [Charrad et al., 2014] and Model posterior distribution given by CU-MSDSp, *Drosophila melanogaster* embryogenesis.

	K	π_1	π_2	π_3	π_4	π_5	π_6	π_7	π_8
Estimates	2	0.44025	0.55975						
	3	0.22825	0.44927	0.32242					
	4	0.18910	0.37559	0.22465	0.21066				
	5	0.18501	0.25013	0.20871	0.17574	0.18030			
	6	0.14642	0.18902	0.15715	0.18185	0.18757	0.13790		
	7	0.15492	0.18249	0.12486	0.14230	0.06795	0.15460	0.17284	
	8	0.12389	0.05843	0.18409	0.12592	0.14291	0.09242	0.10572	0.16647
Realised	2	0.45972	0.54028						
	3	0.24713	0.44955	0.30332					
	4	0.19571	0.37553	0.22314	0.20563				
	5	0.18315	0.25397	0.20462	0.17988	0.17838			
	6	0.14774	0.18968	0.15590	0.16739	0.19432	0.14497		
	7	0.15439	0.18208	0.11521	0.14259	0.07660	0.14975	0.17938	
	8	0.12733	0.06944	0.19646	0.11992	0.14183	0.08602	0.11176	0.14723

Table 14: Summary of estimates and realised values given by the BELMM, *Drosophila melanogaster* embryogenesis, full data set.

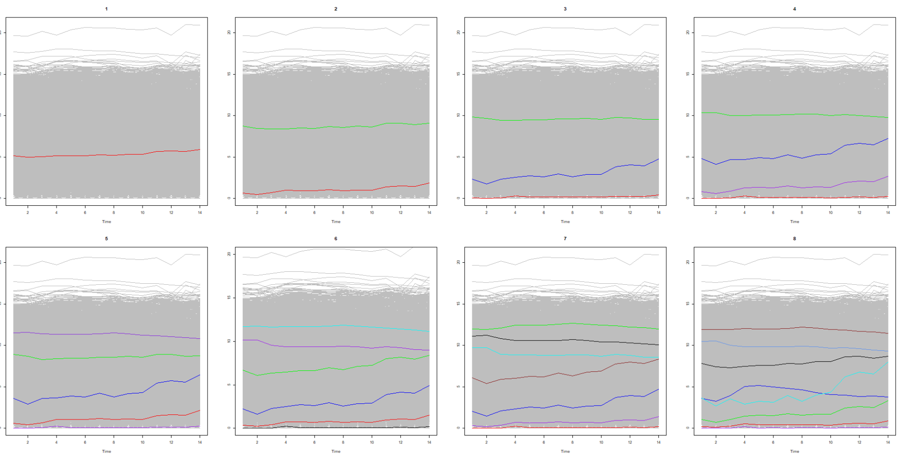

(a) Centres.

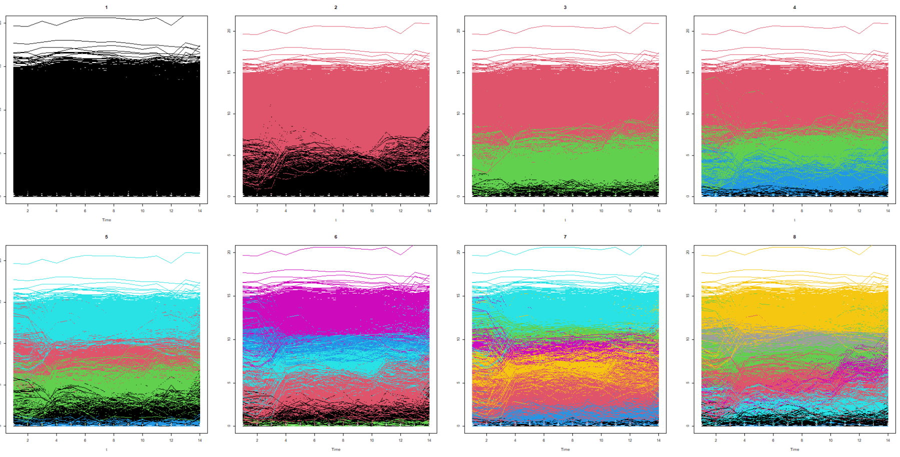

(b) Clusters.

Figure 33: *Drosophila melanogaster* embryogenesis data set; Centres and clusters given by BELMM approach.

Analysis on full data, 2-5 components

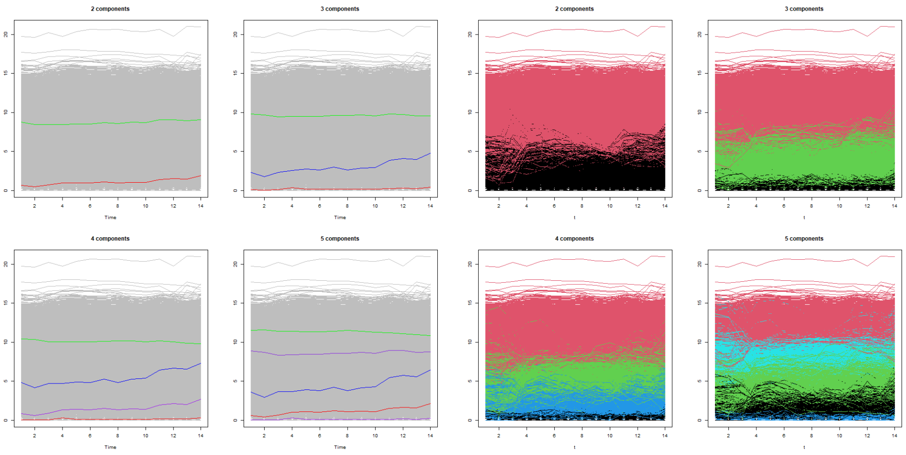

(a) Centres. (b) Clusters.

Figure 34: *Drosophila melanogaster* embryogenesis data set; Centres and clusters given by BELMM approach.

	K	π_1	π_2	π_3	π_4	π_5
Estimates	2	0.44039	0.55961			
	3	0.22818	0.44933	0.32251		
	4	0.18911	0.37570	0.22458	0.21068	
	5	0.18498	0.18035	0.20866	0.17581	0.25010
Realised	2	0.45972	0.54028			
	3	0.24713	0.44955	0.30332		
	4	0.19571	0.37553	0.22321	0.20556	
	5	0.18321	0.17844	0.20462	0.17982	0.25391

Table 15: Summary of results given by the BELMM approach, *Drosophila melanogaster* embryogenesis data set.

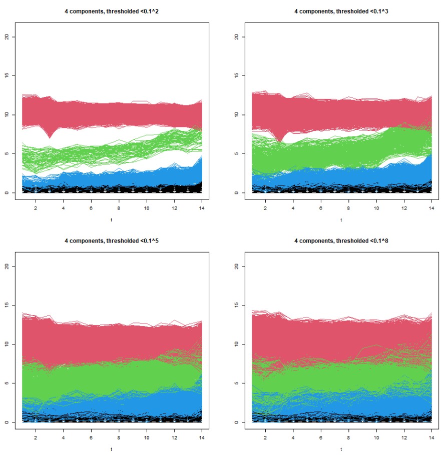

(a) Thresholded clusters.

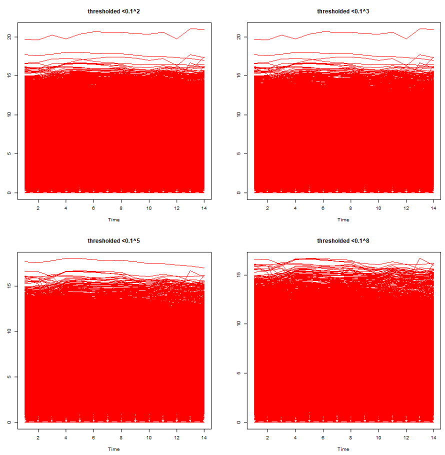

(b) Left-over series.

Figure 35: Thresholded results given by BELMM approach, *Drosophila melanogaster* embryogenesis data set.

	Threshold	1	2	3	4	Thresholded
Realised	0.1^2	2585	1481	66	324	11471
	0.1^3	2884	2374	357	1104	9208
	0.1^5	3113	3523	1239	2327	5725
	0.1^8	3117	4562	2247	2895	3106
Without Thresholding		3117	5981	3555	3274	

Table 16: Summary of thresholded results given by BELMM approach, *Drosophila melanogaster* embryogenesis data set.

5.2 Analysis on random subsets

Here, we show how the BELMM approach can be used to estimate the mixture component centres from random subsets of data.

The sample of 1000 random genes from *Drosophila melanogaster* data is shown in Figure 36a, and a sample of 2000 random genes in Figure 37a. The model posterior distributions are given in Figure 36b and 37b, respectively (*It is not the same figure). The estimated mixture component centres and the resulting clusters for the eight models for the 1000 and 2000 gene samples are shown in Figure 38a and 38b, and 39a and 39b, respectively. Summaries of the results for both samples are given in Table 17 and 18, respectively.

Eight models

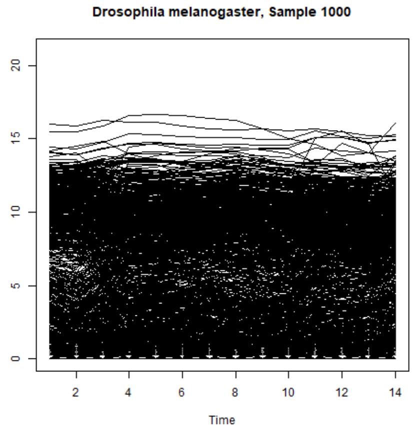

(a) Data.

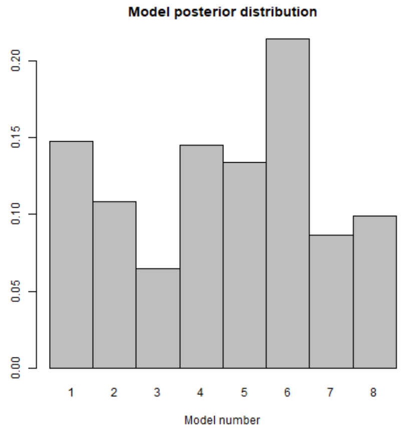

(b) Model posterior distribution

Figure 36: Sample of 1000 genes and the Model posterior distribution given by `CU-MSDSp`, *Drosophila melanogaster* embryogenesis.

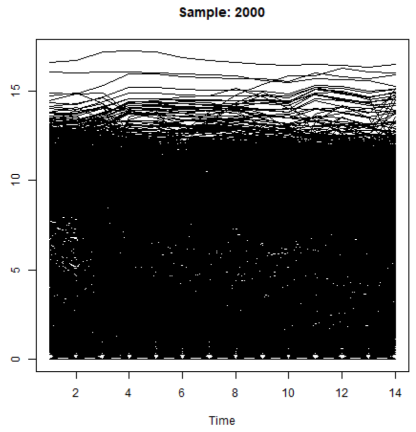

(a) Data.

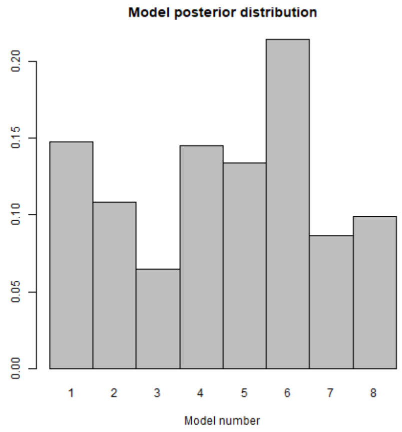

(b) Model posterior distribution.

Figure 37: Sample of 2000 genes and the Model posterior distribution given by `CU-MSDSp`, *Drosophila melanogaster* embryogenesis.

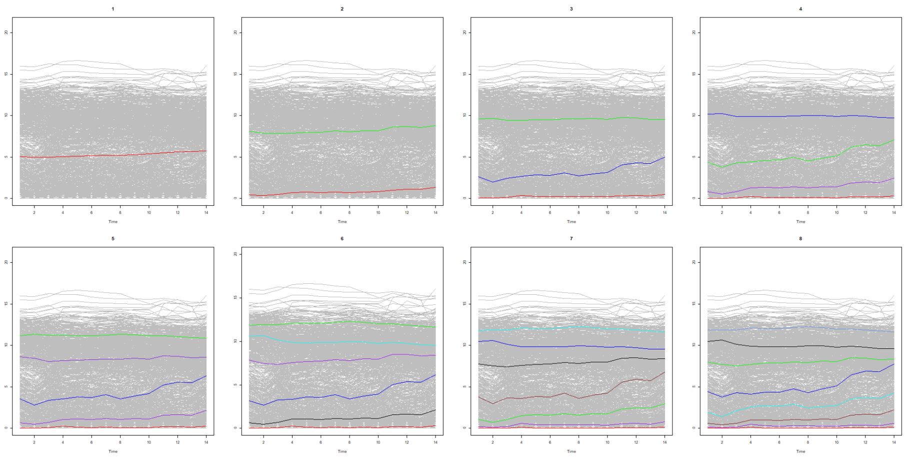

(a) Centres.

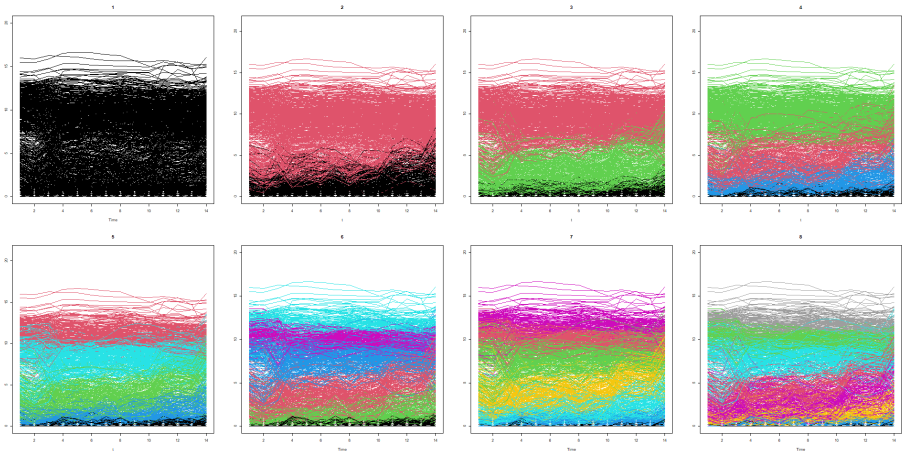

(b) Clusters.

Figure 38: Results for the slow implementations of the model given by the BELMM approach. A sample of 1000, *Drosophila melanogaster* embryogenesis data set.

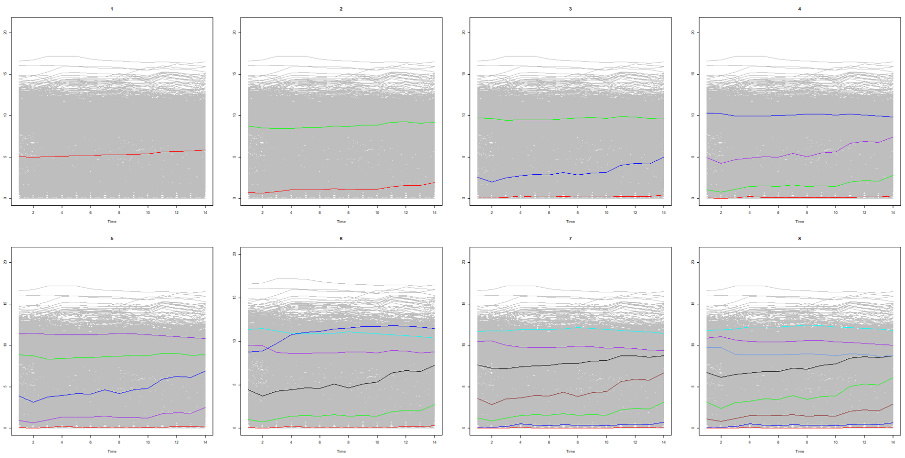

(a) Centres.

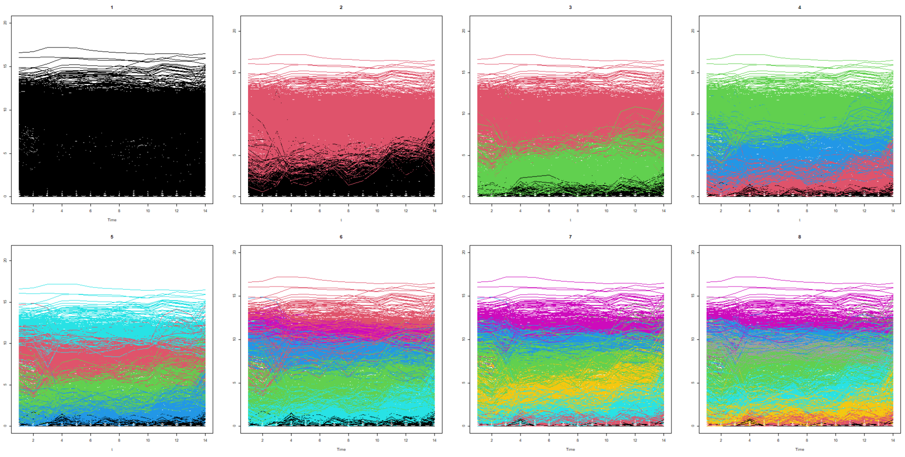

(b) Clusters.

Figure 39: Results for the slow implementations of the model given by the BELMM approach. A sample of 2000, *Drosophila melanogaster* embryogenesis data set.

	K	π_1	π_2	π_3	π_4	π_5	π_6	π_7	π_8
Estimates	2	0.39405	0.60595						
	3	0.25360	0.44148	0.30447					
	4	0.19514	0.22564	0.38305	0.19575				
	5	0.18385	0.19247	0.19914	0.17956	0.24415			
	6	0.18280	0.19071	0.17597	0.18823	0.09945	0.16155		
	7	0.11117	0.16062	0.18463	0.14209	0.14124	0.10006	0.15827	
	8	0.09987	0.10744	0.15799	0.11813	0.17911	0.12586	0.11040	0.09948
Realised	2	0.426	0.574						
	3	0.266	0.442	0.292					
	4	0.200	0.222	0.385	0.193				
	5	0.189	0.190	0.199	0.174	0.248			
	6	0.186	0.191	0.175	0.187	0.104	0.157		
	7	0.108	0.157	0.185	0.140	0.147	0.104	0.159	
	8	0.088	0.108	0.147	0.126	0.186	0.132	0.109	0.104

Table 17: Summary of estimates given by BELMM, *Drosophila melanogaster* embryogenesis, sample of 1000.

	K	π_1	π_2	π_3	π_4	π_5	π_6	π_7	π_8
Estimates	2	0.45394	0.54606						
	3	0.23428	0.43888	0.32654					
	4	0.19545	0.21323	0.37070	0.22000				
	5	0.19163	0.24874	0.19091	0.19385	0.17420			
	6	0.19403	0.06301	0.20244	0.19286	0.20537	0.14162		
	7	0.11477	0.12442	0.18022	0.17049	0.15481	0.10442	0.14996	
	8	0.11404	0.12258	0.15404	0.11621	0.13700	0.07588	0.14318	0.13627
Realised	2	0.471	0.529						
	3	0.2500	0.4405	0.3095					
	4	0.2015	0.2070	0.3715	0.2200				
	5	0.1975	0.2515	0.1895	0.1890	0.1725			
	6	0.2000	0.1155	0.1990	0.1875	0.2005	0.0975		
	7	0.1080	0.1295	0.1920	0.1535	0.1535	0.1100	0.1535	
	8	0.1075	0.1260	0.1695	0.1105	0.1405	0.0825	0.1425	0.1210

Table 18: Summary of estimates given by BELMM, *Drosophila melanogaster* embryogenesis, sample of 2000.

Subsets of 1000 and 2000, 2-5 models, no prior on π_k

The results from the fast and slow implementations are provided for comparison with the results from the eight models; The model posterior distributions are given in Figure 40a and 40b, respectively. The estimated mixture component centres and the resulting clusters for the four models for the 1000 and 2000 gene samples are shown in Figure 41 and 42, and 43 and 44, respectively. Summaries of the results for both samples are given in Table 19 and 21, respectively. For easier comparison, the results from the full data set are provided in Table 20 and 22. The estimated mixture component centres from all the four cases are plotted in one figure over the data in Figure 45.

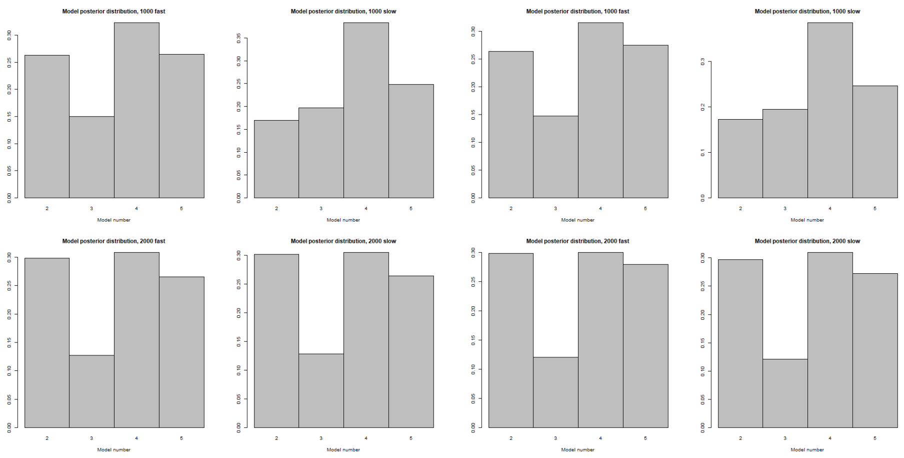

(a) Last 8000. (b) Full chain.

Figure 40: Model posterior distributions given by `CU-MSDSp` for fast and slow implementations of the model. A sample of 1000 and 2000, *Drosophila melanogaster* embryogenesis data set.

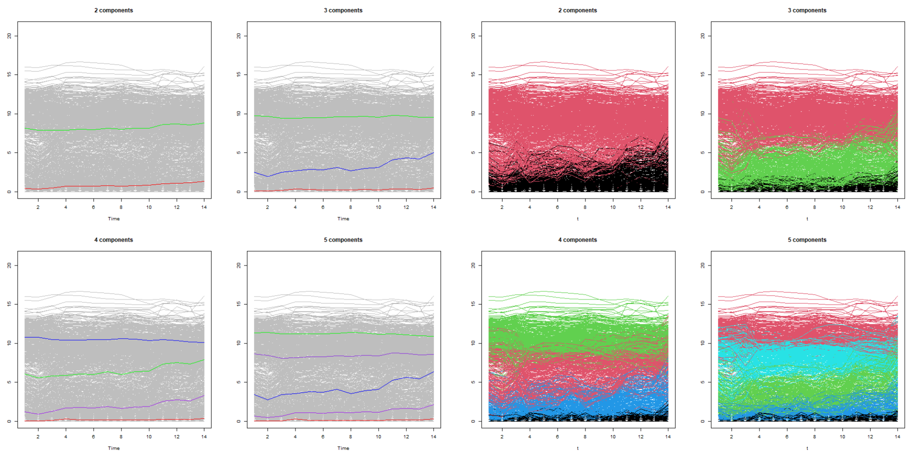

(a) Centres. (b) Clusters.

Figure 41: Results for the fast implementations of the model given by BELMM approach. A sample of 1000, *Drosophila melanogaster* embryogenesis data set.

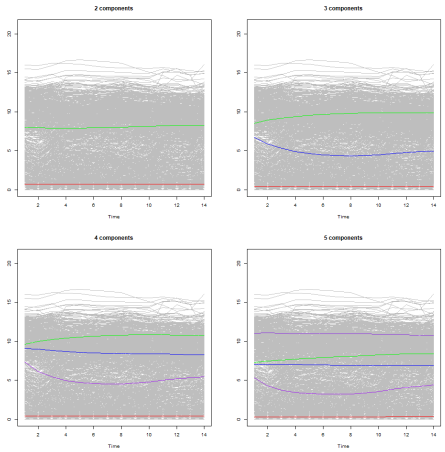

(a) Centres.

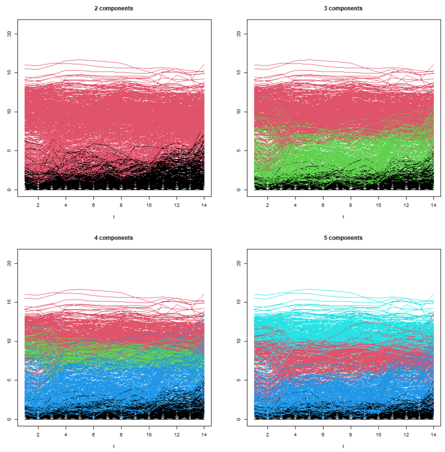

(b) Clusters.

Figure 42: Results for the slow implementations of the model given by the BELMM approach. A sample of 1000, *Drosophila melanogaster* embryogenesis data set.

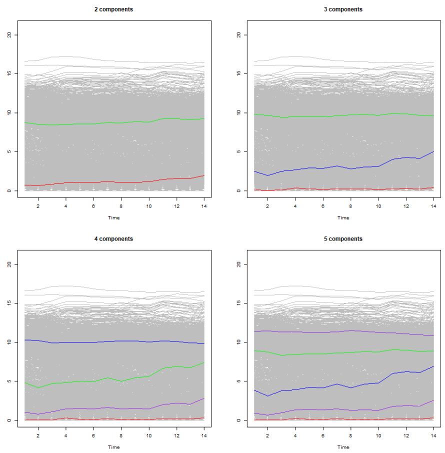

(a) Centres.

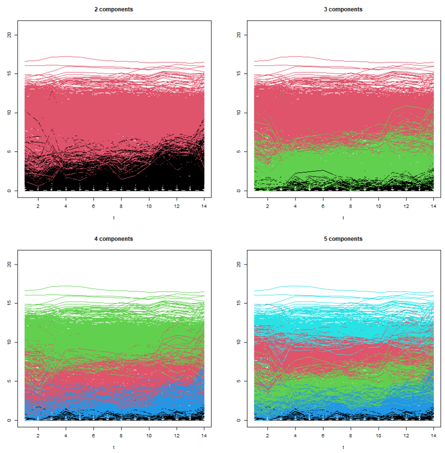

(b) Clusters.

Figure 43: Results for the fast implementations of the model given by the BELMM approach. A sample of 2000, *Drosophila melanogaster* embryogenesis data set.

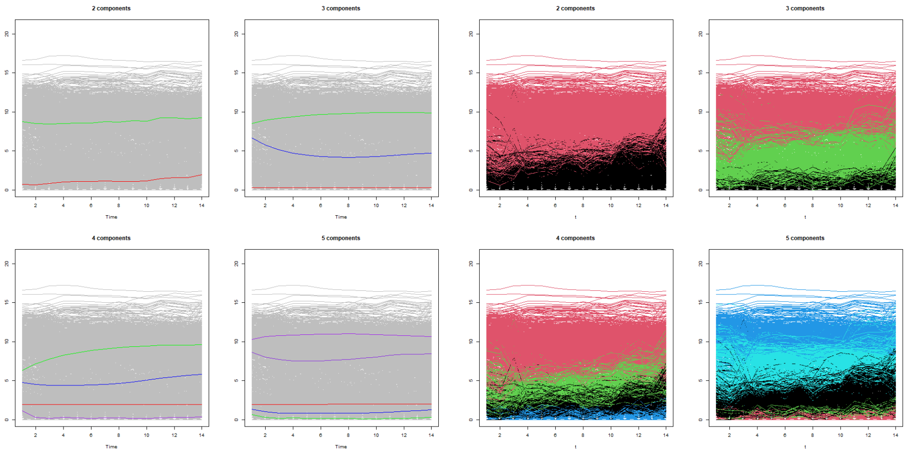

(a) Centres. (b) Clusters.

Figure 44: Results for the slow implementations of the model given by the BELMM approach. A sample of 2000, *Drosophila melanogaster* embryogenesis data set.

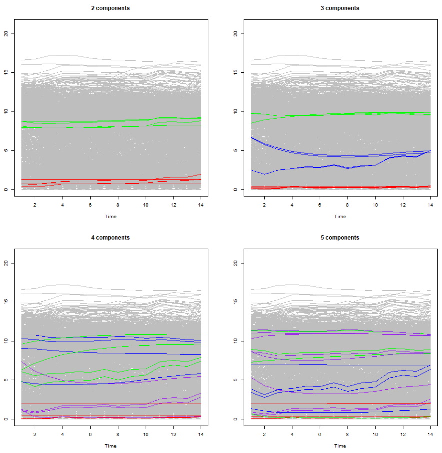

Figure 45: Centres given by the BELMM approach, fast and slow implementations, summary, *Drosophila melanogaster* embryogenesis data set.

Sample: 1000	K	π_1	π_2	π_3	π_4	π_5	Slow	π_1	π_2	π_3	π_4	π_5
Estimated	2	0.39401	0.60599					0.3704	0.6296			
	3	0.25314	0.44169	0.30493				0.29246	0.39773	0.30927		
	4	0.21342	0.24477	0.30686	0.23396			0.29339	0.23896	0.12894	0.33829	
	5	0.18355	0.19067	0.19968	0.17923	0.24594		0.26417	0.20473	0.00927	0.28952	0.23112
Realised	2	0.426	0.574					0.407	0.593			
	3	0.266	0.442	0.292				0.324	0.397	0.279		
	4	0.221	0.244	0.306	0.229			0.327	0.246	0.127	0.300	
	5	0.189	0.189	0.199	0.174	0.249		0.291	0.212	0.004	0.263	0.230

Table 19: Summary of estimates for 2-5 models given by the BELMM approach, *Drosophila melanogaster* embryogenesis, sample of 1000.

	K	π_1	π_2	π_3	π_4	π_5
Estimates	2	0.44039	0.55961			
	3	0.22818	0.44933	0.32251		
	4	0.18911	0.37570	0.22458	0.21068	
	5	0.18498	0.18035	0.20866	0.17581	0.25010

Table 20: Summary of estimates for 2-5 models given by the BELMM approach, *Drosophila melanogaster* embryogenesis full data set.

Sample: 2000	K	π_1	π_2	π_3	π_4	π_5	Slow	π_1	π_2	π_3	π_4	π_5
Estimated	2	0.45385	0.54615					0.45971	0.54029			
	3	0.23419	0.43900	0.32639				0.25852	0.40368	0.33761		
	4	0.19531	0.21926	0.37278	0.21239			0.16847	0.44636	0.15017	0.23462	
	5	0.19159	0.24934	0.19112	0.19372	0.17384		0.22352	0.20074	0.07562	0.22610	0.27345
Realised	2	0.471	0.529					0.4745	0.5255			
	3	0.2500	0.4405	0.3095				0.2945	0.4040	0.3015		
	4	0.2015	0.2185	0.3735	0.2065			0.1660	0.4455	0.1500	0.2385	
	5	0.1970	0.2515	0.1895	0.1895	0.1725		0.2495	0.2030	0.0475	0.2215	0.2785

Table 21: Summary of estimates for 2-5 models given by the BELMM approach, *Drosophila melanogaster* embryogenesis, sample of 2000.

	K	π_1	π_2	π_3	π_4	π_5
Estimates	2	0.44039	0.55961			
	3	0.22818	0.44933	0.32251		
	4	0.18911	0.37570	0.22458	0.21068	
	5	0.18498	0.18035	0.20866	0.17581	0.25010

Table 22: Summary of estimates for 2-5 models given by the BELMM approach, *Drosophila melanogaster* embryogenesis full data set.

5.3 Influence of starting values on the posterior estimates

To study the stability of results under random seeds, we ran our five-component model five times in parallel on a subset of 3000 genes using the `R` included script. We observed that the estimated mixture weights were consistent across the runs, the centres differing slightly in one of the models (Figure 46).

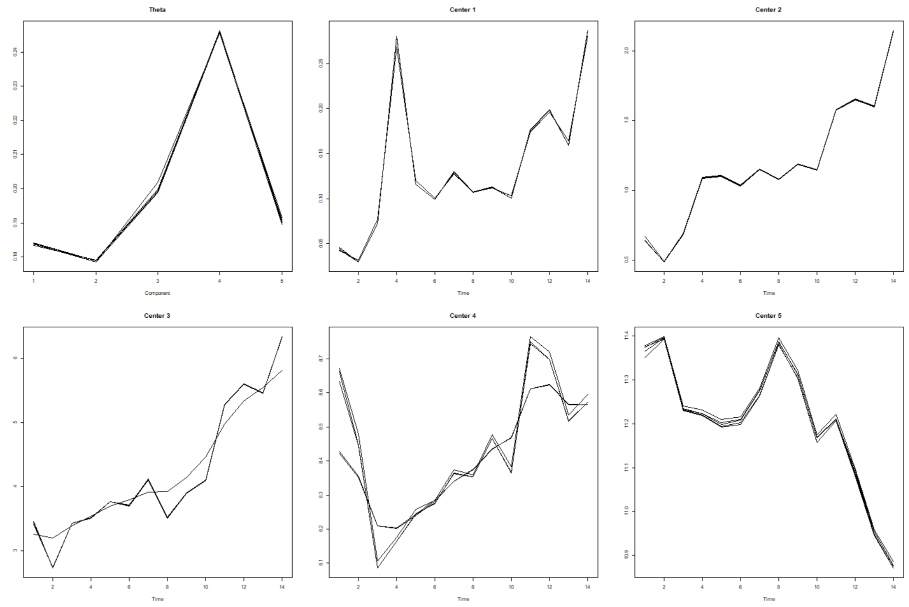

Figure 46: Summary of estimates given by BELMM, fast implementation, *Drosophila melanogaster* embryogenesis data set, sample of 3000.

6 Appendix: Software details

6.1 `BELMM`

For our implementation of the BELMM approach and the tutorial, please refer to the GitHub page at `https://github.com/ollisa/BELMM`. Currently, the RJMCMC is dependent on Stan and CU-MSDSP, of which the latter requires the use of Linux (alternatively Windows subsystem for Linux) or Mac OS. Therefore, we have not made a self-contained `R` package version of the BELMM.

To give an idea of the system requirements, all the models were estimated using a four-core Intel i5-6600 system with 8 GB of RAM for the simulated data sets and for the real data sets with models 2-5 at a time. The WSL tutorial is run on a twelve-core Intel i7-12700 system (using 8 cores for all examples) with 32 GB of RAM. The highest memory usage we have seen during the compilation of the models (8 at a time) by the WSL is a little less than 10 GB, making the total system usage 14 GB with the operating system. If one uses the plotting

option for our models in CU-MSDSp, the software can easily use more than 32 GB of RAM. We do not recommend using the plotting functionality for long time series, such as the French mortality data set, as it can crash the terminal.

To give an example of the runtime, we can consider the 2k sample of the *Drosophila melanogaster* data set. The total times spent for the estimation with the i5 system were 3624,9s for the fast and 17106,1s for the slow. In both cases, 3100s were used for running the RJMCMC. The difference between them is just the slower sampling during the estimation of the models. On the WSL with the i7 system, the fast implementation was done in 1018,3s, and the slow one took 9307,8s for the same data and models. With further optimisation for the model structure (splitting up the for-loops), the slow implementation with four extra models, now 8 in total, takes 2053s on the i7 system for the 2k sample and 7628,7s for the complete data set.

6.2 `R`

We assume the user is familiar with R software and R programming.

6.3 Stan

(Stanr and cmdstan): For installation and a brief example of the basics of Stan software [Stan Development Team, 2020, `http://mc-stan.org/`], we refer the reader to `https://github.com/stan-dev/rstan/wiki/RStan-Getting-Started`.

Some notes on using Stan: Our implementation requires users to write models in the Stan programming language. Writing individual models is easy using R. For small changes, it is also possible to create copies of the files and make the changes using software like Notepad.

The model itself is composed of blocks. The 'Data block' contains information on the data, possible fixed prior values, and the time series data. Making data transformations in Stan using the 'Transformed data' block is also possible. Any sampled model parameters are written in the 'Parameters' block, and any variables dependent on the parameters can be created in the 'Transformed parameters' block for more efficient computation. Model dependencies, such as the prior distributions and their contribution to the Jacobian, are defined in the 'Model' block. Last, the 'Generated quantities' block can generate values based on the sampled parameters, which is optional but used to create the model's log-likelihood for RJMCMC.

Stan uses a JSON-like file format for including the data. In practice, this means writing the data to a list format in `R` and including the dimension of the data and other fixed values, such as the smoothing priors used in our implementation. Many nested models may be required to find the optimal number of components. Including one vector containing the number of components in the Stan data file is beneficial. This reduces the risk of making mistakes in the actual models if the model structure allows this, like in the case of nested models. This can be implemented by including the following changes to the 'data'

block:

```
int number_of_components_model_i = number_of_components[i];
```

However, this is optional, and we just made the changes on a case-by-case basis by hand.

Fast and slow implementations

The difference between the fast and slow implementations is within the sampling statements in the 'model' block. How they work in Stan can be found in `https://mc-stan.org/docs/reference-manual/sampling-statements.html`.

In short, dropping some additive constants when evaluating the incremental log probability makes the calculation faster but may numerically affect the parameter estimates even though the models are mathematically equivalent. Hence, there will be an effect on the model posterior distribution, which depends on the individual models' log-likelihoods. Whether these differences are substantial enough to justify the longer computational time is up to the user to decide.

6.4 CU-MSDSp

For in-depth instructions, please refer to GitHub: `https://github.com/jtchavisIII/CU-MSDSp`. Here are some considerations.

The software CU-MSDSp is used with `bash` (Linux, Mac, and so on. . .). It uses the same JSON format as Stan, so the Stan data file must be written to JSON before the models can be estimated. This can be done in many ways, and we have included a short `R` script that does this.

Installation of the `CU-MSDSp` is detailed on the GitHub page and is done by downloading the included files and installing the required dependencies. After that, running the software works by calling the function in `bash`. Depending on the operating system, there might be some changes needed to the code before it works correctly. For example, the operation on Ubuntu LTS 20.04 required adding a package call to the compiler, but one's experience may vary.

The' config' file includes general parameters for estimating the models. These are the length of warmup 'NBURN' and the collected chain 'NLEN'. The length of the RJMCMC chain is twice that of the collected chain. 'NBURN' should be set so the model with the most mixture components converges. This can be checked by running the model with the most parameters independently in `R`.

Plotting all the posterior distributions for the parameters using the Python file included with `CU-MSDSp` is possible. However, we do not recommend this since the time series models may have 150+ parameters (up to 1000 in some of our examples); this becomes memory intensive fast and could crash the session. Also, it is not strictly necessary since all the models should be convergent at this point. The most important plot is the "Model posterior distribution", which can be plotted using a separate Python script (in the results folder). Plotting

the parameters later using `R` is also possible. Also, manual plotting is necessary if any empty components are found.

The chains on individual models are contained in the 'goldStandardChains' folder. The results from RJMCMC can be found in the 'modelSelection' folder. The run time is in the 'timings' file; any plotted figures are found in the 'pics' folder.

6.5 TMixClust

Quoting Golumbeanu [2020], "TMixClust is a soft-clustering method which employs mixed-effects models with nonparametric smoothing spline fitting and is able to robustly stratify genes by their complex time series patterns. The package has, besides the main clustering method, a set of functionalities assisting the user to visualise and assess the clustering results, and to choose the most optimal clustering solution".

Their model is

$$p(\boldsymbol{X}|\boldsymbol{\theta}) = \sum_{k=1}^{K} \pi_k \mathcal{N}(\boldsymbol{X}|\boldsymbol{\mu}_k, \boldsymbol{\Sigma}_k), \tag{1}$$

where $\boldsymbol{\mu}_k$ is a cubic smoothing spline. The covariance matrix consists of the cluster-specific random effects θ_k and normally distributed random errors θ, so that $\boldsymbol{\Sigma}_k = \mathrm{I} \cdot \theta_k + \mathbf{1} \cdot \theta$. The model with a fixed number of components is estimated using the EM algorithm. At the moment, they do not implement any model selection functionality.

To overcome the EM algorithms' limitation of finding only the local maxima, the package can run the estimation procedure multiple times (e.g. 10, 20, ...) to find a solution with the highest likelihood. They can do clustering stability analysis by calculating the Rand index to measure how well the different runs agree with the best-fitting solution. However, for unknown reasons, their implementation in the function "analyse_stability" does not work correctly for the French mortality data.

6.6 Software versions

Here, we list versions of some notable software.

On Windows 11: `R` 4.2.1 (2022-06-23 ucrt), rstan 2.26.13, rstantools 2.2.0.
For running CU-MSDSp on Ubuntu 20.04 LTS: CmdStan 2.30.0, Open MPI 4.1.1, GNU Scientific Library 2.6.
For running CU-MSDSp on Windows 11 through WSL: Ubuntu 22.04 LTS, Python 3.10.6, scipy 1.11.1, matplotlib 3.7.2, seaborn 0.12.2, b. Open MPI 4.1.2, GNU scientific library 2.35, cmdstan 2.22.

References

Olivier Cappé, Christian P. Robert, and Tobias Rydén. Reversible jump, birth-and-death and more general continuous time Markov chain Monte Carlo sam-

plers. *Journal of the Royal Statistical Society. Series B (Statistical Methodology)*, 65(3):679–700, 2003.

John Taylor Chavis, Amy Louise Cochran, and Christopher James Earls. Cumsdsp: A flexible parallelized reversible jump Markov chain Monte Carlo method. *SoftwareX*, 14:100664, 2021.

Zoé van Havre, Nicole White, Judith Rousseau, and Kerrie Mengersen. Overfitting Bayesian mixture models with an unknown number of components. *PLoS One*, 10(7):e0131739, 2015.

Aritra Guha, Nhat Ho, and Xuanlong Nguyen. On posterior contraction of parameters and interpretability in Bayesian mixture modeling. *Bernoulli (Andover.)*, 27(4), 2021.

Monica Golumbeanu, Sébastien Desfarges, Céline Hernandez, Manfredo Quadroni, Sylvie Rato, Pejman Mohammadi, Amalio Telenti, Niko Beerenwinkel, and Angela Ciuffi. Proteo-Transcriptomic dynamics of cellular response to HIV-1 infection. *Sci Rep*, 9(1):213, 2019.

Monica Golumbeanu. *TMixClust: Time Series Clustering of Gene Expression with Gaussian Mixed-Effects Models and Smoothing Splines*, 2020. R package version 1.10.0.

Malika Charrad, Nadia Ghazzali, Véronique Boiteau, and Azam Niknafs. Nbclust: An R package for determining the relevant number of clusters in a data set. *Journal of Statistical Software*, 61(6):1–36, 2014.

Stan Development Team. RStan: the R interface to Stan, 2020. R package version 2.21.2.
